# Alterations in the gut microbiome and metabolic profile in rats acclimated to high environmental temperature

**DOI:** 10.1111/1751-7915.13772

**Published:** 2021-02-23

**Authors:** Yang Cao, Ying Liu, Qingyang Dong, Tao Wang, Chao Niu

**Affiliations:** ^1^ Department of Environmental Medicine Tianjin Institute of Environmental and Operational Medicine Tianjin 300050 China

## Abstract

Heat acclimation (HA) is the best strategy to improve heat stress tolerance by inducing positive physiological adaptations. Evidence indicates that the gut microbiome plays a fundamental role in the development of HA, and modulation of gut microbiota can improve tolerance to heat exposure and decrease the risks of heat illness. In this study, for the first time, we applied 16S rRNA gene sequencing and untargeted liquid chromatography–mass spectrometry (LC‐MS) metabolomics to explore variations in the gut microbiome and faecal metabolic profiles in rats after HA. The gut microbiota of HA subjects exhibited higher diversity and richer microbes. HA altered the gut microbiota composition with significant increases in the genera *Lactobacillus* (a major probiotic) and *Oscillospira* alongside significant decreases in the genera *Blautia* and *Allobaculum*. The faecal metabolome was also significantly changed after HA, and among the 13 perturbed metabolites, (S)‐AL 8810 and celastrol were increased. Moreover, the two increased genera were positively correlated with the two upregulated metabolites and negatively correlated with the other 11 downregulated metabolites, while the correlations between the two decreased genera and the upregulated/downregulated metabolites were completely contrary. In summary, both the structure of the gut microbiome community and the faecal metabolome were improved after 28 days of HA. These findings provide novel insights regarding the improvement of the gut microbiome and its functions as a potential mechanism by which HA confers protection against heat stress.

## Graphical abstract

Evidence indicates that the gut microbiome plays a fundamental role in the development of HA. In this study, we applied 16S rRNA gene sequencing and untargeted liquid chromatography‐mass spectrometry (LC‐MS) metabolomics to explore variations in the gut microbiome and fecal metabolic profiles in rats after HA. Our study demonstrates for the first time that HA has a significant effect on the gut microbiome and fecal metabolome, and this may be a potential mechanism by which HA confers protection against heat stress.

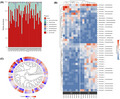

## Introduction

Hot environmental conditions increase physiological strain, attenuate performance capabilities and increase the occurrence of heat illness (Zurawlew *et al*., 2018). Heat acclimation (HA) is a ‘within lifetime’ reversible phenotypic adaptation that occurs during repeated exposure to a hot environment (Horowitz, 2007, 2014, 2016). HA is known to result in numerous positive physiological adaptations that contribute to improved thermoregulation, reduce the risk of heat illness and improve thermal tolerance and human performance in extreme heat (Périard *et al*., 2015; Casa, 2018). Many studies have revealed that human individuals after HA can be characterized by increased sweating efficiency and plasma volume, decreased exercising and resting core temperature, improvements in cardiovascular stability, and whole‐body and skeletal muscle metabolism (Febbraio *et al*., 1994; Nielsen, 1998; Lorenzo *et al*., 2010; Périard *et al*., 2015; Buono *et al*., 2018). Moreover, some studies have found that rodents such as mice and rats exhibit similar physiological responses to HA, including reduced proinflammatory responses, lower heart rats and slower warming during thermal stress (Sareh *et al*., 2011; Yang *et al*., 2017; Yi *et al*., 2017; Bittencourt *et al*., 2020). A general cellular response to heat stress is the induction of heat shock proteins (HSPs), which help to protect against heat‐related illness. We and others have shown that HA leads to a marked upregulation of basal levels of HSP70 and HSP72 in humans and animals (Maloyan *et al*., 1999; Sareh *et al*., 2011; Gibson *et al*., 2016; Yi *et al*., 2017; Nava and Zuhl, 2020). These elevated HSPs improved tolerance to heat stress by reducing inflammatory responses. Current research on HA, however, has focused almost exclusively on physiological and molecular responses, but the molecular mechanisms underlying these responses are still largely unknown.

Microbes that reside in the human gut play a key role in host health and disease, and studies of the gut microbiota have attracted much attention and provide interesting new perspectives and research avenues (Schmidt *et al*., 2018). Hyperthermia or heat stress can adversely disrupt the intestinal mucosa and augment intestinal permeability by destroying the tight junctions of epithelial cells, which results in lipopolysaccharide (LPS) and endotoxin translocation from the intestinal lumen to the circular system, potentially causing heat illnesses such as heat stroke (Dokladny *et al*., 2015; Armstrong *et al*., 2018; Karl *et al*., 2018). Several studies on animals have documented that heat stress may negatively influence gut microbiota, including reduced intestinal microbial diversity and a decreased abundance of probiotics (such as *Lactobacillus* and *Bifidobacteria*) (Chen *et al*., 2018; He *et al*., 2019; Shi *et al*., 2019).

Growing evidence indicates that elevated intracellular HSP can improve heat tolerance, increase resistance to endotoxin translocation and protect from oxidative stress and inflammation by maintaining the tight junctions of intestinal epithelial cells (Kuennen *et al*., 2010; Amorim *et al*., 2015; Arnal and Lallès, 2016). It has been established that dietary fibre, prebiotics and probiotics have beneficial effects on human health by regulating gut microbiota homeostasis (Arnal and Lallès, 2016; Plovier *et al*., 2017). Specifically, *Bacillus subtilis* has been used for the prevention of heat stress by maintaining intestinal permeability and microbial structure, as well as reducing bacterial translocation (Moore *et al*., 2014; Sorokulova *et al*., 2016).

Meanwhile, gut microbial metabolic activities play a critical role in maintaining host homoeostasis and health and are associated with a variety of diseases (Visconti *et al*., 2019). For example, short‐chain fatty acids (SCFAs), fatty acids produced by gut microbial fermentation of indigestible foods, are the main energy source of colonocytes, regulators of cell proliferation and differentiation, and making them anti‐inflammatory agents (Parada Venegas *et al*., 2019). On the other hand, some gut microbial products can act as toxins to host tissues and may result in disease (Louis *et al*., 2014). These findings suggest that the gut microbiome and its metabolic activities play a fundamental role in the development of HA. However, to our knowledge, no studies have explored the association of the gut microbiota with HA.

In this study, we applied 16S rRNA gene sequencing and untargeted liquid chromatography–mass spectrometry (LC‐MS) to investigate the effects of HA on the gut microbiome and the faecal metabolic profiles of rats. This study illustrates a new strategy for advancing our understanding of the biological mechanism of HA.

## Results

### Effects of HA on core body temperature and weight

To confirm that HA had a positive effect on health, the rectal temperature (Tre), the gold standard for measuring core body temperature (Richmond *et al*., 2015; Mazgaoker *et al*., 2017), was assessed and compared between HA and control (CR) subjects. Consistent with previous studies, it was observed that the Tre profile of HA subjects significantly increased in response to the first two weeks after heat exposure, followed by a steady and slow decrease and ultimately return to the same level as before heat exposure (Fig. S1A) (Yi *et al*., 2017). HA subjects grew more slowly than CR subjects (Fig. S1B), and the body weight of HA subjects was significantly (*P* < 0.001, Wilcoxon sum test) lower than CR subjects after our HA experiment had finished.

### Quality control of 16S rRNA microbiome profiling

To assess the changes in the gut microbiota during HA, the gut microbiota were sequenced 1 day before heat exposure (day 0), and at days 14 and 28 after heat exposure. From our 16 S rRNA sequencing (V3‐V4 region), a total of 4 062 872 reads were obtained, and 3 824 443 reads remained after quality filtering, corresponding to a mean of 79 675 reads per sample (ranging from 60 593 to 82 017). Sequencing‐based rarefaction curves of all samples revealed that the sequencing depth was sufficient to describe each associated gut microbial community (Fig. S2B). A total of 1387 operational taxonomic units (OTUs) were identified, which has a minimum count of 0.00005 of total reads across samples. As we expected, no significant differences were found between CR and HA on day 0 by comparing the number of OTUs (1038 and 1071 OTUs; *P* = 0.6, Wilcoxon sum test; Fig. S2A, D), alpha diversity (all *P* > 0.05, Wilcoxon sum test; Fig. S3A) and beta diversity (Fig. S3B, C). The number of OTUs was significant different between CR and HA on day 28 (1207 and 1279 OTUs respectively; *P* = 0.014, Wilcoxon sum test; Fig. S2A, F) rather than on day 14 (1313 and 1295 OTUs respectively; *P* = 0.19, Wilcoxon sum test; Fig. S2A, E). Interestingly, the number of OTUs of CR subjects increased at the first stage (from day 0 to day 14) and then decreased to close to the number on day 0 at the second stage (from day 14 to day 28). Furthermore, all the differences in the number of OTUs in CR rats were not significant (Fig. S2C). We speculated that the gut microbiome of CR subjects changed due to the new living environment (at the first stage) and then recovered to their initial states after a certain period of adaptation (at the second stage).

### HA induces changes in the gut microbiota

**Fig. 1:**
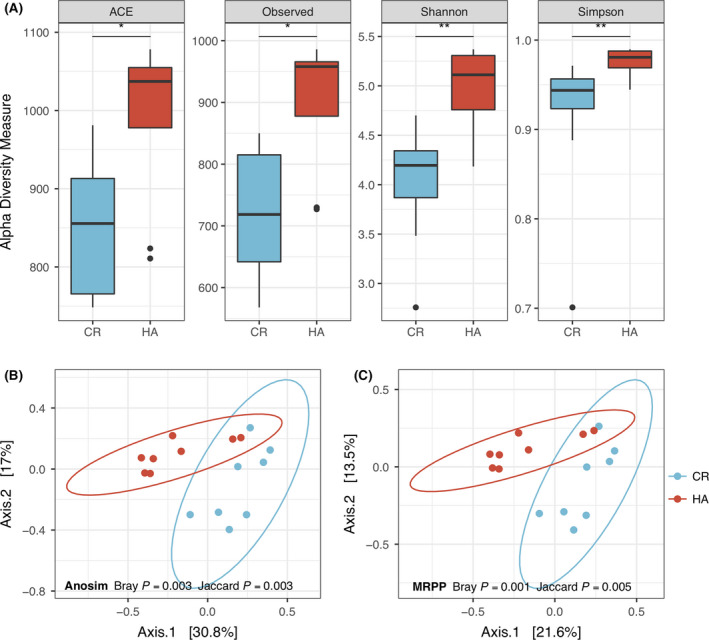
Diversity analysis on day 28. A. Alpha diversity assessed by richness (ACE, Observed) and diversity (Shannon, Simpson). Box plots showing significantly different alpha diversity between HA and CR subjects by Wilcoxon rank sum test. Beta diversity assessed by principal coordinate analysis (PCoA) based on the Bray–Curtis (B) and Jaccard (C) distances. Anosim and multi‐response permutation procedure (MRPP) tests show statistically significant differences between HA and CR groups. *P* values: *P < 0.05, **P < 0.01.

We then compared the composition of the gut microbiome between different groups by measuring microbial alpha and beta diversities. Alpha diversity, measured by four indices, ACE, Observed, Shannon and Simpson, showed a significant increase in the HA subjects compared to the CR subjects on day 28 (Fig. 1A, all *P* < 0.05, Wilcoxon sum test), and no significant differences were observed in alpha diversity indices on day 14 (all *P* > 0.05, Wilcoxon sum test; Fig. S4A). Principal coordinate analysis (PCoA), based on Bray–Curtis and Jaccard dissimilarity, showed that HA subjects were clearly separated from CR subjects on day 28 (all *P* < 0.05, Anosim and multi‐response permutation procedure (MRPP) analysis, for Bray and Jaccard distances respectively) (Fig. 1B, C). Moreover, no significant differences were found for samples from day 14 (Fig. S4B, C).

Since population differences in the gut microbiota were only observed between HA and CR subjects on day 28, only subjects on day 28 were considered in subsequent analyses. To explore the taxa that were relevant to HA, we performed differential abundance analysis at the phylum and genus levels comparing between these two groups.

**Fig. 2:**
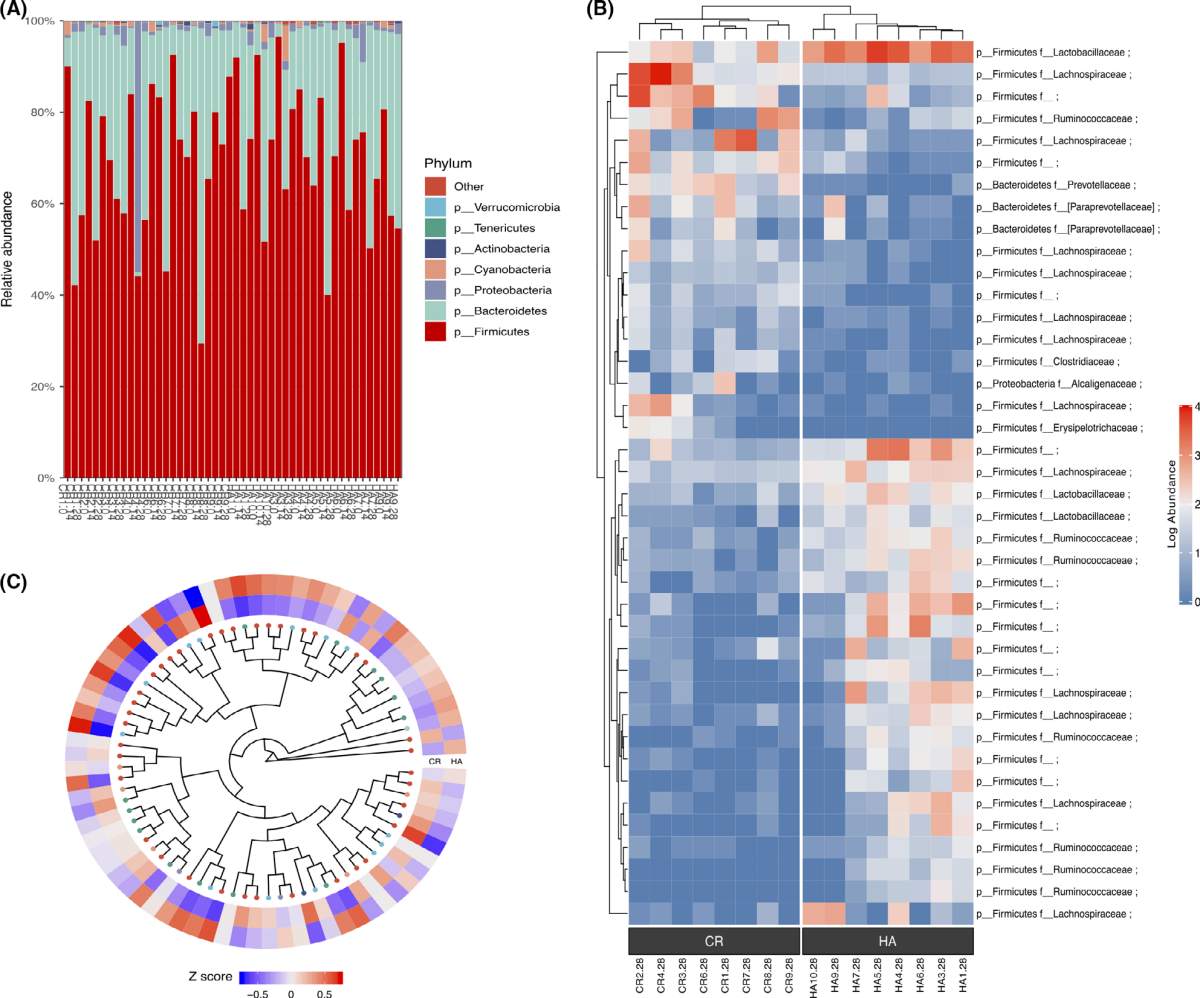
Composition analysis on day 28. A. Relative abundance at the phylum level. B. Heat map based on hierarchical clustering analysis shows the relative abundance of the top 40 different representative taxa between HA and CR on day 28. Differential abundance analysis was performed using DESeq2. The taxonomy of taxa included phylum and family. C. Heatmap tree showing genera significantly different in HA compared to those in CR, and their phylogenetic relationships on day 28. The abundance profiles are expressed by z‐scores.

At the phylum level, the dominant bacteria in all samples were *Firmicutes*, followed by *Bacteroidetes, Proteobacteria*, *Cyanobacteria* and *Actinobacteria* (Fig. 2A; see also Fig. S4D). There were subtle but not significant differences in the bacterial community composition between HA and CR subjects at the phylum level (all *P* > 0.05, Wilcoxon sum test; Fig. S4D and Table S1). The genus level analysis showed that the genera *Lactobacillus* (a major probiotic) and *Oscillospira* were significantly increased, while the genera *Blautia* and *Allobaculum* were significantly decreased in HA subjects (Table S2). The genera abundance distributions in different groups were quite different (Fig. 2C).

**Fig. 3:**
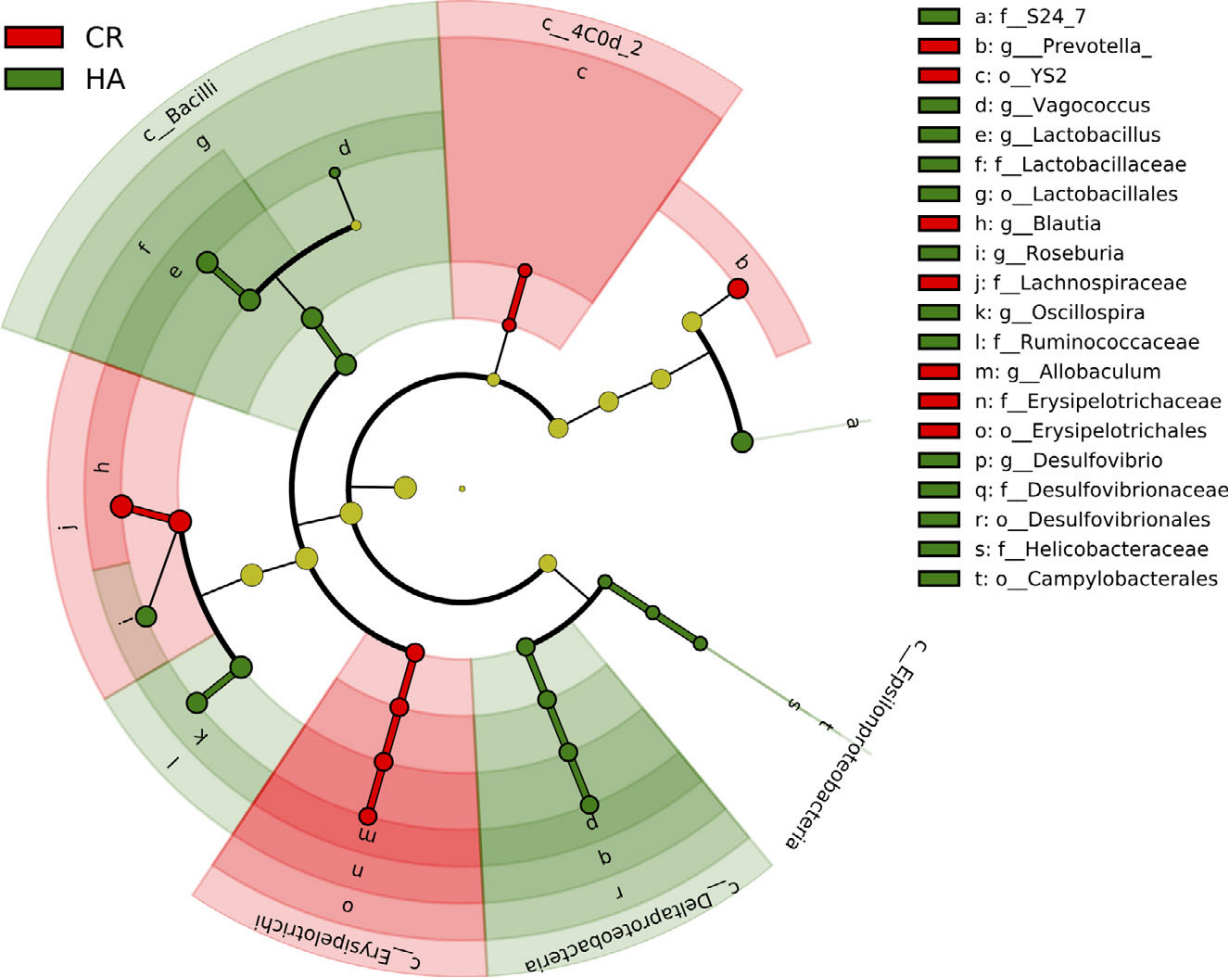
Cladogram by LEfSe analysis showing the biomarker taxa associated with HA. Green indicates taxa enriched in the HA group, while red indicates the taxa enriched in the CR group.

We next performed differential abundance analysis using DESeq2 (Love *et al*., 2014), a method using negative binomial GLM to obtain maximum likelihood estimates between two conditions, so that we could more accurately explore the bacterial taxa that contributed to the differentiation of the gut microbiota composition between HA and CR groups. Then, a heat map from hierarchical clustering analysis based on the top 40 different taxa was used to summarizes the intersample changes in HA and CR groups. We found a clear separation of the gut microbiota from HA and CR subjects (Fig. 2B; see also Table S3). Differentially abundant taxa (Table S4) were further confirmed by LEfSe analysis (Segata *et al*., 2011). The results are shown in Fig. 3, and 20 taxa, including 8 genera, were significantly different between the HA and CR groups. Consistent with the above results, the genus *Lactobacillus* was enriched in HA subjects.

### HA harbours a modified microbial ecological network

**Fig. 4:**
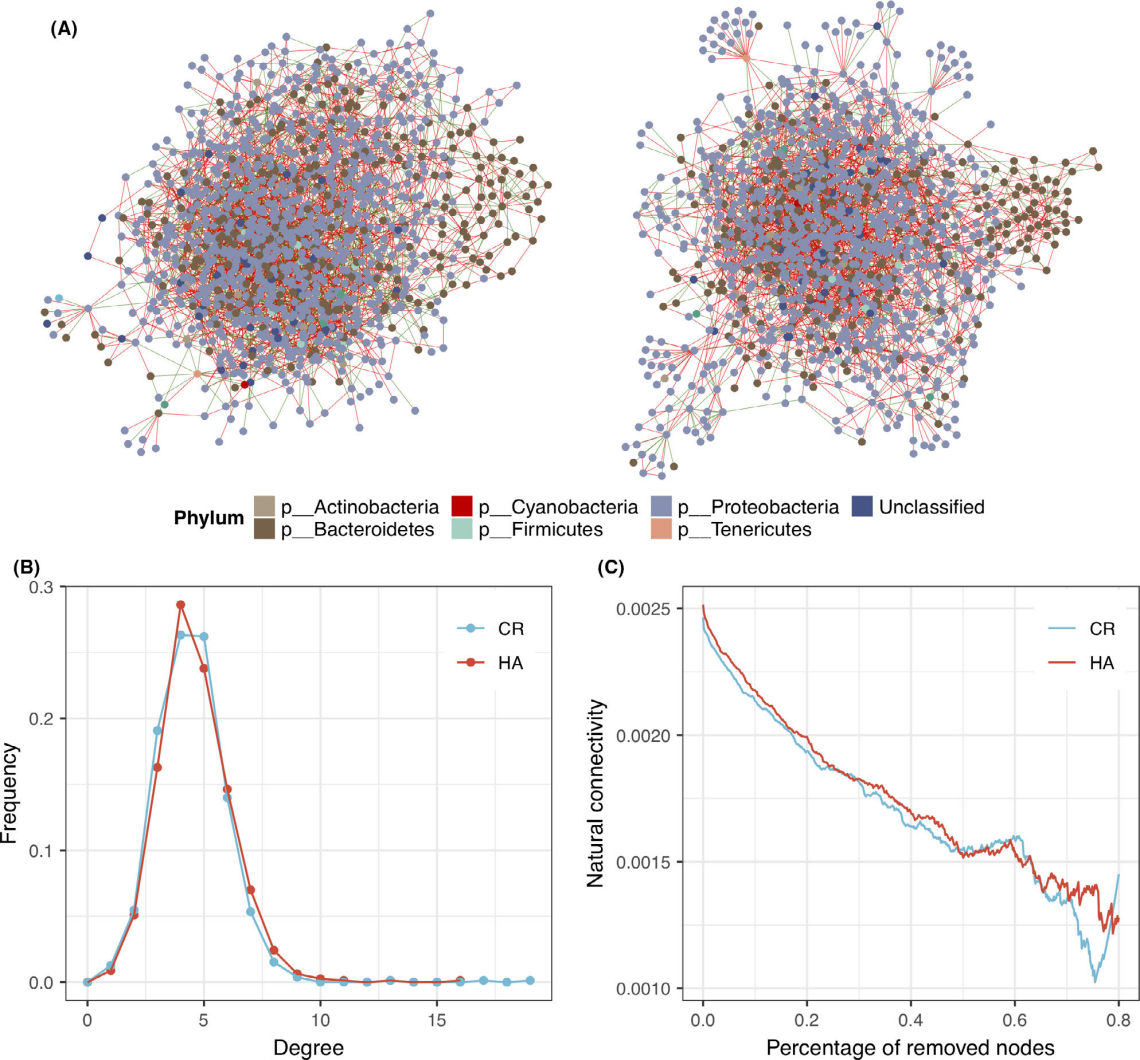
Ecological networks inferred using SPIEC‐EASI in the HA (A) and CR (B) groups. Nodes represent OTUs and are coloured according to phyla; red edges represent positive correlations, and green edges represent negative correlations. C. Degree distributions of the two networks were similar. D. Natural connectivity was used to measure the robustness of networks by sequentially removing high degree nodes. The result showed that the HA network was more robust than the CR network.

To examine the changes in the HA microbial community structure and how HA affects the microbial interactions, we utilized Sparse InversE Covariance estimation for Ecological Association (SPIEC‐EASI) (Kurtz *et al*., 2015) to infer two microbial ecological networks in HA and CR subjects. In general, there were more positive correlations than negative correlations in both ecological networks (Fig. 4A, B). Correlations between two OTUs in the same genus tended to be positive, whereas correlations between two OTUs in different genera involved both positive and negative correlations.

The degree distributions of the vertices were similar between the two networks (Fig. 4C). We first compared the degrees of OTUs in the four significantly different genera, *Blautia*, *Oscillospira*, *Lactobacillus* and *Allobaculum*. The degree of *Lactobacillus* in the HA network was significantly lower than in the CR network (*P* = 0.0396, Wilcoxon sum test), while the degrees of the OTUs in other three genera were similar in the two networks (Fig. S5). We then compared the robustness of these networks against attacks by sequentially removing hubs (nodes with high degree centrality). The results showed that the HA network was more robust than the CR network by removing high degree nodes (Fig. 4D).

### HA induces changes in the metabolite profiles in faecal samples

**Fig. 5:**
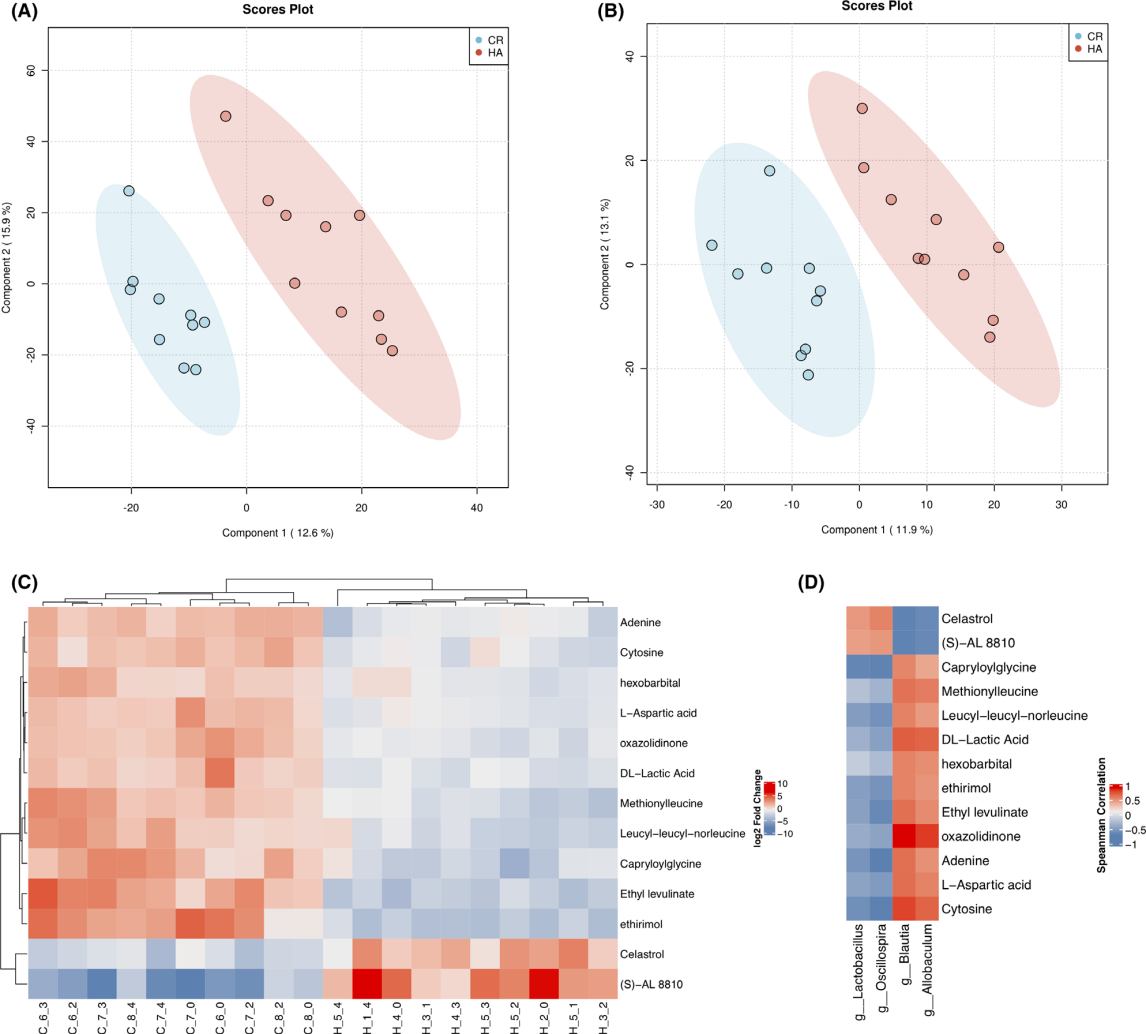
PLS‐DA score plot of faecal metabolome in the ESI+ (A) and ES‐ modes (B). C. Heatmap tree showing faecal metabolites significantly different in HA compare to those in CR. D. Correlations between perturbed gut‐bacterial families and altered faecal metabolites. Spearman’s rank correlation coefficients and *P* values for the correlations of faecal bacteria and their metabolites.

Since 16S rRNA sequencing revealed that the gut microbiome had significantly changed after 28 days of HA, we further examined whether the faecal metabolome was perturbed after 28 days of HA using untargeted LC‐MS in both positive ion (ES+) and negative ion (ES‐) modes. A total of 2588 and 1715 features were identified in the ES + and ES‐ modes respectively. The partial least squares discriminant analysis (PLS‐DA) score plots showed a clear separation between the HA and CR groups (Fig. 5A, B). Ten‐fold cross‐validation was employed to evaluate the quality of these two PLS‐DA components, giving an R2 of 0.901 (0.935) and Q2 of 0.403 (0.349) in the ES + and ES‐ modes respectively. Overall, the faecal metabolome was significantly changed after HA.

To identify the differential metabolites associated with HA, we calculated fold changes (FCs), *P* values and PLS‐DA variable importance in the projection (VIP) scores for all metabolic features in HA vs. CR. In total, 9 and 34 significant metabolic features (FC ≥ 2 or ≤ 0.5, *P* ≤ 0.05, and VIP > 2) were found in the ESI + and ESI‐ analysis modes respectively. As we expected, there was a clearly separation in the faecal metabolome from HA and CR subjects using hierarchical clustering based on differential metabolites (Fig. 5C).

To further understand these metabolic changes at the pathway level, we performed pathway enrichment analysis using MetaboAnalystR (Chong *et al*., 2019). A total of nine metabolic pathways were significantly changed after HA, including glycine, serine and threonine metabolism, lysine degradation, tyrosine metabolism, aminoacyl‐tRNA biosynthesis, steroid hormone biosynthesis, nicotinate and nicotinamide metabolism, glycerolipid metabolism, amino sugar and nucleotide sugar metabolism (Table S5). Finally, there were 13 significant differential metabolites by overlapping between the putatively annotated features in our pathway enrichment analysis and the significant metabolic features found in univariate statistical analysis. Of these 13 metabolites, two metabolites were upregulated in HA subjects, including (S)‐AL 8810 and celastrol. Additionally, 11 other metabolites were downregulated, including hexobarbital, cytosine, L‐aspartic acid, adenine, oxazolidinone, DL‐lactic acid, leucyl‐leucyl‐norleucine, methionylleucine, capryloylglycine and ethyl levulinate ethirimol. Celastrol has been shown to be capable of inducing heat shock factor 1 (HSF1)‐mediated HSP genes, such as HSP30 and HSP70, which play an important role in protection against heat stress (Walcott and Heikkila, 2010; Ma *et al*., 2015). Targeted metabolomics was employed to validate the expression of celastrol, and the results corroborated the increased level of celastrol after HA (Fig. S6).

### Correlation between differential gut microbes and metabolites

Since both gut microbiota and metabolites were perturbed after HA, we utilized correlation analysis to explore the functional correlations between the four perturbed genera and the 13 altered faecal metabolites using Spearman’s correlation coefficient. As shown in Fig. 5D, the increases in the genera *Lactobacillus* and *Oscillospira* were positively correlated with the two upregulated metabolites (S)‐AL 8810 and celastrol and negatively correlated with the other 11 downregulated metabolites. However, the two decreased genera showed a contrary correlation. These data indicated that the gut microbiota contributes to changes in the faecal metabolome.

## Discussion and conclusions

Heat acclimation results in physiological adaptations that can improve heat tolerance and performance, and reduce physiological strain and the risk of heat illness in hot conditions. Previous studies have revealed that heat stress and heatstroke increased gut permeability and impaired barriers to bacteria, leading to a significant perturbation in the gut microbial community in animals (Vargas and Marino, 2016; Armstrong *et al*., 2018; He *et al*., 2019; Zhu *et al*., 2019). In addition, probiotics, such as *Bacillus subtilis*, may protect people or animals against the effects of heat stress, allowing them to recover more quickly to full health by enhancing gut integrity and improving the gut microbiome (Moore *et al*., 2014; Song *et al*., 2014). Based on these studies, we inferred that gut microbiota play an important role in the response to HA. Our study aimed to investigate the impacts of HA on the gut microbiome and metabolome using 16S rRNA gene sequencing and LC‐MS.

To establish an HA model, we held rats in a chamber at 35 ± 1°C and 60 ± 5% humidity for 120 min per day for 28 days and explored the effect of HA on core body temperature and weight. The core body temperature was significantly decreased after HA, consistent with previous findings (Shmeeda *et al*., 2002; Périard *et al*., 2015). We then determined whether HA influenced the faecal microbiota in rats by comparing the microbial communities in HA and CR subjects. Microbiome diversity analysis showed that population differences in the gut microbiota could only be observed between HA and CR subjects on day 28, rather than on day 0 or day 14 after treatment. HA subjects exhibited higher diversity and richer microbes compared with CR subjects on day 28. It has been proposed that rich and diverse microbiomes are more stable and more healthy (Lozupone *et al*., 2012). These findings suggested a clear HA in rats could be established after 28 days of heat exposure, similar to previous research (Umschweif *et al*., 2013; Yi *et al*., 2017).

To further explore more accurately the bacterial taxa that contributed to the differentiation of the gut microbiota composition between different groups, we performed differential analysis at the phylum and genus levels. At the phylum level, *Firmicutes*, *Bacteroidetes*, *Proteobacteria*, *Cyanobacteria* and *Actinobacteria* were the five dominant phyla, in agreement with previous studies (Hu *et al*., 2016; Khan *et al.,* 2016). There were, however, subtle but not significant differences in the bacterial community compositions. Our genus level analysis indicated that *Lactobacillus* and *Oscillospira* were significantly increased in HA subjects. It has been reported that *Lactobacillus* is very important for mediating innate and adaptive immune defences against microbial pathogens and preventing stress‐induced dysfunction of the colonic epithelial barrier function in adult animals (Gareau *et al*., 2010). Bacterial taxa showing significantly different abundances were further confirmed by LEfSe analysis. Probiotics such as *Lactobacillus* may someday be selectively prescribed to attenuate heat stress‐induced intestinal permeability and provide cytoprotection against heat stress. The level of *Oscillospira* showed a positive association with human health, while a decrease in *Oscillospira* may lead to diseases that involve inflammation (Gophna *et al*., 2017).

To examine how HA affects the microbial interactions in the gut microbiome, we inferred two ecological networks using the SPEIC‐EASI algorithm. There were more co‐occurrence correlations than co‐exclusion correlations in both networks. Consistent with a previous study, correlations between OTUs in the same genus tended to be positive, and correlations between OTUs in different genera were involved in both positive and negative correlations (Leung *et al*., 2018). In addition, we showed that the HA network displayed more robustness against simulated attack by sequentially removing high degree nodes, compared to our CR network.

Next, an untargeted metabolomics approach, LC‐MS, was used to reveal the effect of 28 days of HA on the faecal metabolome. In total, 43 metabolites were changed as a result of HA using differential analysis. Pathway enrichment analysis revealed that metabolites involved in lysine degradation, aminoacyl‐tRNA biosynthesis, and glycine, serine and threonine metabolism were significantly altered after HA. Finally, 13 significant differential metabolites were obtained by overlapping the putatively annotated metabolites in enriched pathways and the significant metabolic features found by differential analysis, of which (S)‐AL 8810 and celastrol were significantly increased. Previous studies have shown that celastrol, a natural product derived from the roots of *Tripterygium wilfordii*, exhibits antioxidant, anti‐inflammatory, antiobesity and anticancer activities and is a pharmacologically active regulator of the heat shock response (Westerheide *et al*., 2004; Kannaiyan *et al*., 2011; Liu *et al*., 2015). Thus, we infer that celastrol makes an important contribution to the protect effect of HA in hot environments.

Finally, Spearman’s correlation analysis was conducted to explore the relationships between significantly changed genera and metabolites. The results showed that the two increased genera were positively correlated with the 2‐upregulated metabolites and negatively correlated with the 11‐downregulated metabolites, while the correlations between two decreased genera and the upregulated/downregulated metabolites were completely contrary. These findings suggest that gut microbiota modification induced by HA was correlated with faecal metabolites.

This study did have limitations. To characterize the changes in the gut microbiota during HA, faecal samples were collected and sequenced on the day 0, 14 and 28 after heat exposure. However, HA is a longer‐term chronic process. The gut microbiomes at additional time points may have been explored for more reliable and precisely results using both comparative and longitudinal analyses. Although 16S rRNA gene sequencing technology is highly useful for microbiome studies, it provides low resolution at the species level and poor discriminatory power for some genera (Janda and Abbott, 2007). Ideally, whole‐metagenome shotgun sequencing can provide higher strain‐level resolution and more accurate information related to the composition and function of a microbial community. Additionally, although we found significant correlations between differential gut microbiota and differential metabolites, more work needs to be performed in future studies to explore the mechanism of these correlations. Finally, findings in this study were correlational and do not support causal relationship between gut microbiota and HA, indicating a lack of sufficient evidence to distinguish the gut microbiota as a cause or consequence of HA. Future studies using strategies such as faecal microbiota transplantation (FMT) are needed to elucidate the underlying causality. Nevertheless, our study demonstrates for the first time that HA has a significant effect on the gut microbiome and faecal metabolome, and this may be a potential mechanism by which HA confers protection against heat stress.

## Experimental procedures

### Animals and experimental conditions

This study was conducted in strict accordance with the recommendations in the Guide for the Care and Use of Laboratory Animals of the US National Institutes of Health. The protocol was approved by the Ethics Committee of Laboratory Animals, Tianjin Institute of Environmental and Operational Medicine. Adult SD rats (8 weeks of age, male, 190–210 g) were fed laboratory chow *ad libitum*. All rats were held under a 12‐h light/dark cycle at 23 ± 1°C and 50 ± 10% humidity. Rats were randomly divided into two groups (8 animals per group), heat acclimated (HA) and normothermia (CR). Rats in the CR group were maintained at an ambient temperature of 23 ± 1°C, and rats in the HA group were kept in a chamber under 35 ± 1°C and 60 ± 5% humidity for 120 min per day for 28 days. Tre was measured every two days, and the body weight was measured weekly during heat exposure.

### Sample collection and processing

Fresh faeces from each animal were collected on day 0, 14 and 28 after heat exposure. The samples were immediately frozen in liquid nitrogen and then stored at −80°C until DNA extraction.

### 16S rRNA gene sequencing

Total faecal DNA was extracted using the CTAB/SDS method. DNA concentration and purity were measured using agarose gels (1%). DNA was diluted to 1 ng µl^‐1^ using sterile water. 16S rRNA genes of V3‐V4 region were amplified with primers (515F and 806R) and were tagged with a barcode. All PCR reactions were carried out in 30 µl reactions, containing 15 µl of Phusion® High‐Fidelity PCR Master Mix (New England Biolabs, Ipswich, MA, USA), 10 ng template DNA and 0.2 µM of each primer. Thermal cycling began with an initial denaturation for 1 min at 98°C, followed by 30 cycles of denaturation for 10 s at 98°C, annealing for 30 s at 50°C and elongation for 30 s at 72°C, followed by extension for 5 min at 72°C.

The same volume of PCR products and 1 × loading buffer containing SYB green were mixed and detected by agarose gel electrophoresis (2%). The PCR products were mixed, and then, the mixture of PCR products was purified with the GeneJETTM Gel Extraction Kit (Thermo Scientific, Waltham, MA, USA). Sequencing libraries were generated using Ion Plus Fragment Library Kit (Thermo Scientific, Waltham, MA, USA) following the manufacturer's instructions. The quality of libraries was determined using the Qubit@ 2.0 Fluorometer (Thermo Scientific, Waltham, MA, USA). These high‐quality libraries were sequenced on the Ion S5TM XL platform and single‐end 400 (600) bp raw reads were generated at Novogene (Tianjin, China).

### Metagenomic data analysis

Based on each unique barcode, single‐end reads were assigned to different samples. To obtain high‐quality clean reads, these assigned reads were cleaned by removing adaptors, primers and low quality reads using Cutadapt (Martin, 2011). Chimeric sequences were detected by aligning clean reads to the Greengenes database (v 13_5) (DeSantis *et al*., 2006) using the UCHIME algorithm (Edgar *et al*., 2011), and the effective reads were obtained by removing chimeric sequences. QIIME1 (v1.9.1) (Caporaso *et al*., 2010) was used to cluster the effective reads into OTUs based on 97% similarity using Uparse (Edgar, 2013). Representative sequences for each OTU were screened for further annotation. OTUs were then annotated using the Greengenes database (v13_5). OTUs at low abundance (fraction of the total OTU observation lower than 0.00005) were discarded.

Four alpha diversity indices were calculated to measure the diversity within samples. Two indices, the Chao and the ACE estimator, were taken to measure the microbial community richness, while two indices, the Shannon and Simpson index, were taken to measure the microbial community diversity. Comparative analysis of the group‐specific α‐diversity indices was performed using a Wilcoxon rank sum test. Beta diversity was used to evaluate the diversity among samples. First, taxa abundance was normalized according to the sequence number of the lowest sample. Then, the beta diversity was assessed by PCoA using Bray–Curtis and Jaccard distances. Beta diversity was tested using Anosim and MRPP. The functions *adonis* and *mrpp* from the vegan package (Dixon, 2003) were taken to calculate pairwise distances and 999 permutations. Both alpha diversity and beta diversity in our samples were calculated with phyloseq (McMurdie and Holmes, 2013) and were visualized with ggplot2 (Wickham, 2016).

Heatmap tree was used to compare the abundances (expressed by *z*‐scores) of all taxa between HA and CR subjects, and this was visualized using ComplexHeatmap (Gu *et al*., 2016). A Wilcoxon rank sum test with Benjamin and Hochberg false discovery rate (FDR) correction was used for differential abundance analysis at the phylum and genera levels, and an FDR‐corrected *P* value < 0.1 was considered significantly different. Moreover, differentially abundant taxa were identified using DESeq2 (Love *et al*., 2014). A heat map based on hierarchal clustering analysis was used to show the relative abundance of the top 40 different taxa, which was visualized with ggtree (Yu *et al*., 2017). Statistically significant bacterial differences (LDA > 2, *P* < 0.05) associated to HA were explored using linear discriminant analysis (LDA) effect size (LEfSe) (Segata *et al*., 2011).

### Microbial ecology network construction

To minimize the interference from low confidence OTUs, OTUs that were less than 100 reads over all samples or those that were present in less than 30% of samples were filtered out. The remained OTUs were selected for inferring a microbial ecology network using SPIEC‐EASI (Kurtz *et al*., 2015). The SpiecEasi parameters were set to method = “mb”, lambda.min.ratio = 1e^‐2^, nlambda = 10, pulsar.params = list(rep.num = 100). Only correlations whose magnitudes were above 0.05 were considered as significant correlations. Networks were visualized using Cytoscape (v3.7.1) (Shannon *et al*., 2003). Two general network properties, including degree distribution and natural connectivity, were determined for network robustness comparison.

### Faecal metabolomics profiling

#### Sample preparations

Faecal samples from day 28 after heat exposure from the two groups were taken for LC‐MS. Samples were homogenized, and 200 μl of each sample was dried using a vacuum. Then the faecal sample and prechilled methanol (400 μl) were mixed by vortexing. The mixtures were incubated on ice for 5 min and then were centrifuged at 15 000 rpm at 4°C for 5 min. Some of the supernatant was diluted to its final concentration containing 53% methanol by LC‐MS grade water. The samples were subsequently transferred to a fresh Eppendorf tube and then were centrifuged again at 15 000 rpm at 4°C for 10 min. Finally, the supernatant was injected into the LC‐MS/MS system analysis.

#### HPLC‐MS experiments

LC‐MS analyses were performed using a Vanquish UHPLC system (Thermo Fisher, Waltham, MA, USA) coupled with an Orbitrap Q Exactive series mass spectrometer (Thermo Fisher, Waltham, MA, USA). Samples were injected onto a Hyperil Gold column (100 × 2.1 mm, 1.9 μm) using a 16‐min linear gradient at a flow rate of 0.2 ml min^‐1^. The eluents for the positive polarity mode were (A) 0.1% formic acid in water and (B) methanol. The eluents for the negative polarity mode were (A) 5 mm ammonium acetate, pH 9.0 and (B) methanol. The solvent gradient was set as follows: 2% B, 1.5 min; 2–100% B, 12.0 min; 100% B, 14.0 min; 100–2% B, 14.1 min; 2% B, 17 min. The Q‐Exactive series mass spectrometer was operated in positive/negative polarity mode with spray voltage of 3.2 kV, a capillary temperature of 320°C, a sheath gas flow rate of 35 arb and an aux gas flow rate of 10 arb.

#### Data analysis

The raw data files generated by UHPLC‐MS/MS were processed using Compound Discoverer 3.1 (CD3.1; Thermo Fisher) to perform peak alignment, peak picking and quantitation for each metabolite. The main parameters were set as follows: retention time tolerance, 0.2 min; actual mass tolerance, 5 ppm; signal intensity tolerance, 30%; signal/noise ratio, 3; and minimum intensity, 100 000. After that, peak intensities were normalized to the total spectral intensity. The normalized data were used to predict the molecular formula based on additive ions, molecular ion peaks and fragment ions. Then, peaks were matched with the mzCloud (https://www.mzcloud.org/), mzVault and MassList databases to obtain accurate qualitative and relative quantitative results. All metabolites were annotated using the KEGG (http://www.genome.jp/kegg/), HMDB (http://www.hmdb.ca/) and Lipidmaps (http://www.lipidmaps.org/) databases. PLS‐DA was used to characterize the metabolic changes induced by HA, and pathway enrichment analyses were performed. We applied univariate analysis (t‐test) to calculate the statistical significance (*P* value). The metabolites with *P* < 0.05, FC ≥ 2 or FC ≤ 0.5, and VIP > 2 obtained from PLS‐DA analysis were considered to be significant differential metabolites. For clustering heat map generation, the data were normalized using z‐scores of the intensity areas of differential metabolites and were visualized using ComplexHeatmap. Pathway activity analysis was predicted with both mummichog and gene set enrichment analysis (GSEA) methods using MetaboAnalystR (Chong *et al*., 2019).

### Targeted metabolomics of celastrol

Two hundred mg of each faecal sample and 1 ml of ethyl acetate were mixed by vortexing. The mixtures were incubated on ice for 30 min and were then centrifuged at 12 000 rpm at 4°C for 10 min. Then, 800 µl of supernatant was extracted and concentrated to dryness in vacuum at room temperature. The residue was reconstituted with 200 μl of methanol and filtered using a 0.22‐μm filter membrane. 10 μl of the resulting solution was aspirated for analysis.

The analysis was performed using a Shimadzu LC30A coupled with a Hybrid Quadrupole‐TOF LC/MS/MS Mass Spectrometer (AB SCIEX, Framingham, MA, USA) in the positive mode. A waters ACQUITY UPLC Xbridge C18 Column (2.5 µm, 2.1 mm × 150 mm) was used with a flow rate at 0.2 ml min^‐1^ and a column temperature of 55°C. The eluents were (A) 0.1% FA in water and (B) acetonitrile. The gradient was set as follows: 5% B at 0 min, 50% B at 7 min, 100% B at 10 min, 100% B at 15 min, 5% B at 16 min and 5% B at 18 min.

### Correlation analysis between the gut and microbiota and faecal metabolome

Spearman’s correlation analysis was performed between significant changed gut microbiota and faecal metabolites using R.

## Funding Information

This study was supported by the National Natural Science Foundation of China (81901914) and the Tianjin Institute of Environmental and Operational Medicine (BWS17J031).

## Conflict of interest

The authors declare no conflicts of interest.

## Supporting information


**Fig. S1**. Mean rectal temperature (A) and body weight (B) during 28 days of heat exposure.

**Fig. S2**. Quality control of 16S rRNA V3‐V4 reads. Number of OTUs (A) after quality filtering on day 0, 14, and 28. Wilcoxon test was used to compare CR and HA. (B) Rarefaction curves for all samples with the X axis representing the number of sequences and the Y axis representing the number of observed taxa. (C) The number of OTUs from the CR group on day 0, 14, and 28. Venn diagram showing the number of OTUs exclusively identified in each group on day 0 (D), 14 (E), and day 28 (F). *P* value: *P < 0.05; ns, no significance *P* > 0.05.

**Fig. S3**. Diversity analysis on day 0. (A) Alpha diversity assessed by richness (ACE, Observed) and diversity (Shannon, Simpson). Boxes represent the interquartile ranges, and the inside black plots represent the median and circles are outliers. *P* values are from Wilcoxon rank sum test. Beta diversity assessed by principal coordinate analysis (PCoA) based on the Bray‐Curtis (B) and Jaccard (C) distances. *P* values are from Wilcoxon rank sum test. *P* values: ns, no significance *P* > 0.05.

**Fig. S4**. The degrees of OTUs in the four significant different genera of inferred ecological networks. *P* values are from Wilcoxon rank sum test. *P* value: *P < 0.05; ns, no significance *P* > 0.05.

**Fig. S5**. Targeted metabolomics profiling of celastrol. *P* values are from Wilcoxon rank sum test. *P* value: *P < 0.05.

**Fig. S6**. Diversity analysis on day 14. (A) Alpha diversity assessed by richness (ACE, Observed) and diversity (Shannon, Simpson). Boxes represent the interquartile ranges, and the inside black plots represent the median and circles are outliers. *P* values are from Wilcoxon rank sum test. Beta diversity assessed by principal coordinate analysis (PCoA) based on the Bray‐Curtis (B) and Jaccard (C) distances. Significant *P*‐values of Anosim and multi‐response permutation procedure (MRPP) between groups emphasize the differences in microbial community structure. (D) Relative abundance of bacterial phyla. *P* values: ng, no significance *P* > 0.05.

**Table S1**. Wilcoxon rank‐sum test comparison of bacterial relative abundances at the phylum level.
**Table S2**. Wilcoxon rank‐sum test comparison of bacterial relative abundances at the genus level.
**Table S3**. Significantly different OTUs between HA and CR subjects which identified using DESeq2.
**Table S4**. Differentially abundant taxa between HA and CR subjects which identified using LEfSe.
**Table S5**. Significantly changed pathways after HA. Pathway activity analyses were predicted with both mummichog and GSEA methods using MetaboAnalystR.

## Data Availability

Raw data for 16S rRNA amplicon sequencing and faecal metabolome are available upon request. The code and all analysis results can be found at https://github.com/yiluheihei/ha_gm.
