# Supplementary figures and images for: Alterations in the gut microbiome and metabolic profile in rats acclimated to high environmental temperature
Source: Microb Biotechnol. 2021 Feb 23;15(1):276–88. doi: 10.1111/1751-7915.13772 (PMC8719808; doi:10.1111/1751-7915.13772)

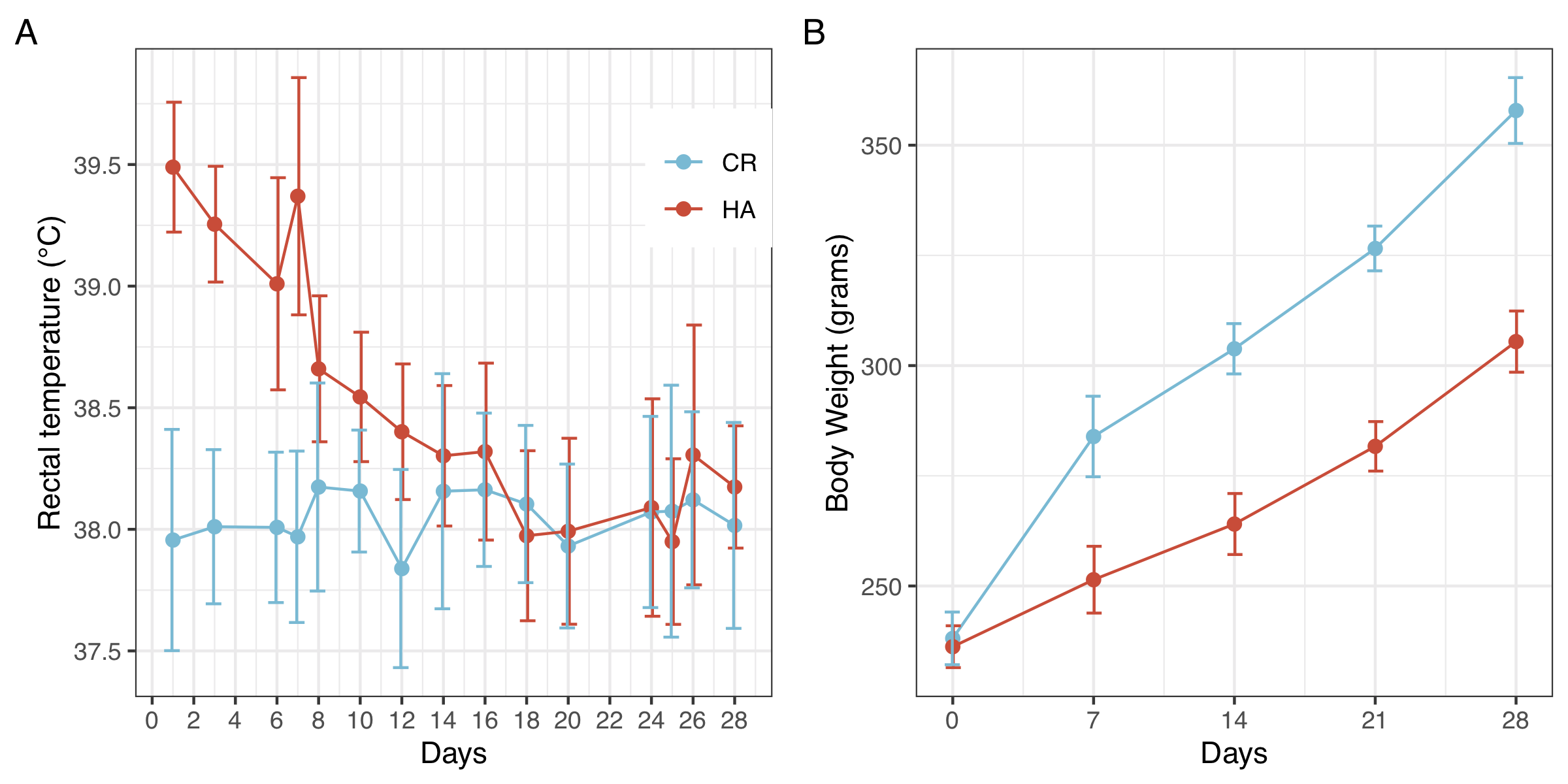

Supplement: Supplementary file 1 — Fig. S1. Mean rectal temperature (A) and body weight (B) during 28 days of heat exposure. [file MBT2-15-276-s001.tiff]

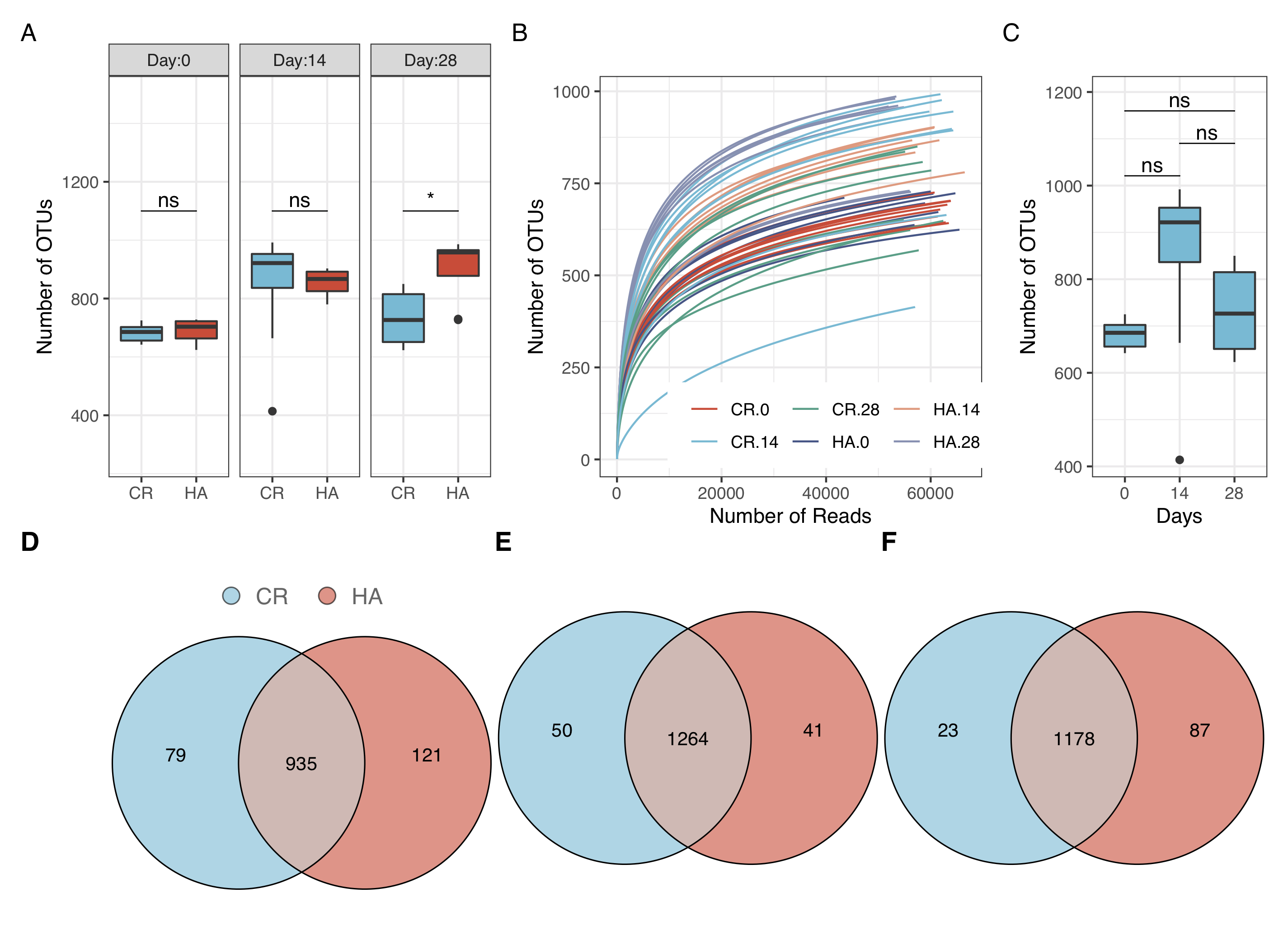

Supplement: Supplementary file 2 — Fig. S2. Quality control of 16S rRNA V3‐V4 reads. Number of OTUs (A) after quality filtering on day 0, 14, and 28. Wilcoxon test was used to compare CR and HA. (B) Rarefaction curves for all samples with the X axis representing the number of sequences and the Y axis representing the number of observed taxa. (C) The number of OTUs from the CR group on day 0, 14, and 28. Venn diagram showing the number of OTUs exclusively identified in each group on day 0 (D), 14 (E), and day 28 (F). P value: *P < 0.05; ns, no significance P > 0.05. [file MBT2-15-276-s003.tiff]

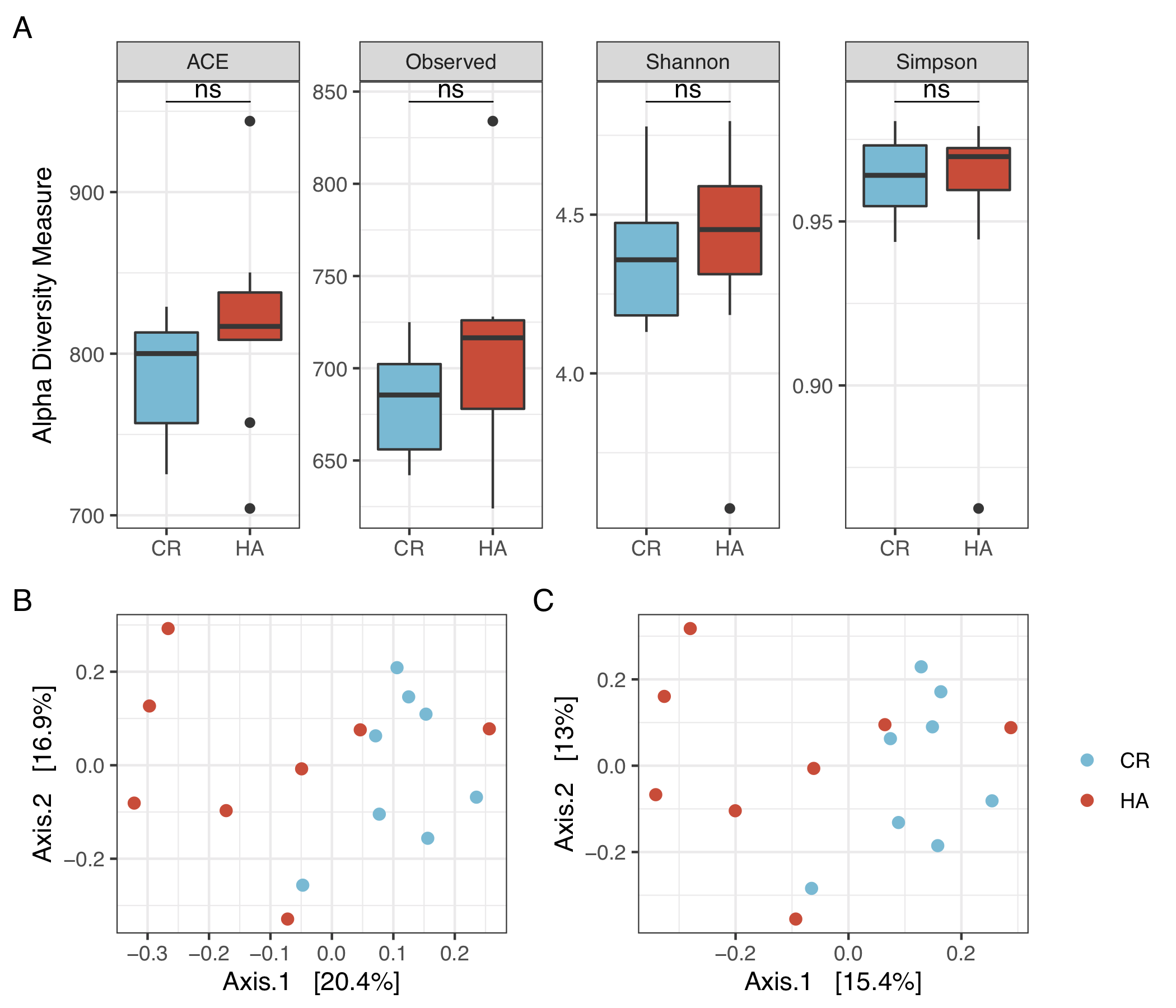

Supplement: Supplementary file 3 — Fig. S3. Diversity analysis on day 0. (A) Alpha diversity assessed by richness (ACE, Observed) and diversity (Shannon, Simpson). Boxes represent the interquartile ranges, and the inside black plots represent the median and circles are outliers. P values are from Wilcoxon rank sum test. Beta diversity assessed by principal coordinate analysis (PCoA) based on the Bray‐Curtis (B) and Jaccard (C) distances. P values are from Wilcoxon rank sum test. P values: ns, no significance P > 0.05. [file MBT2-15-276-s006.tiff]

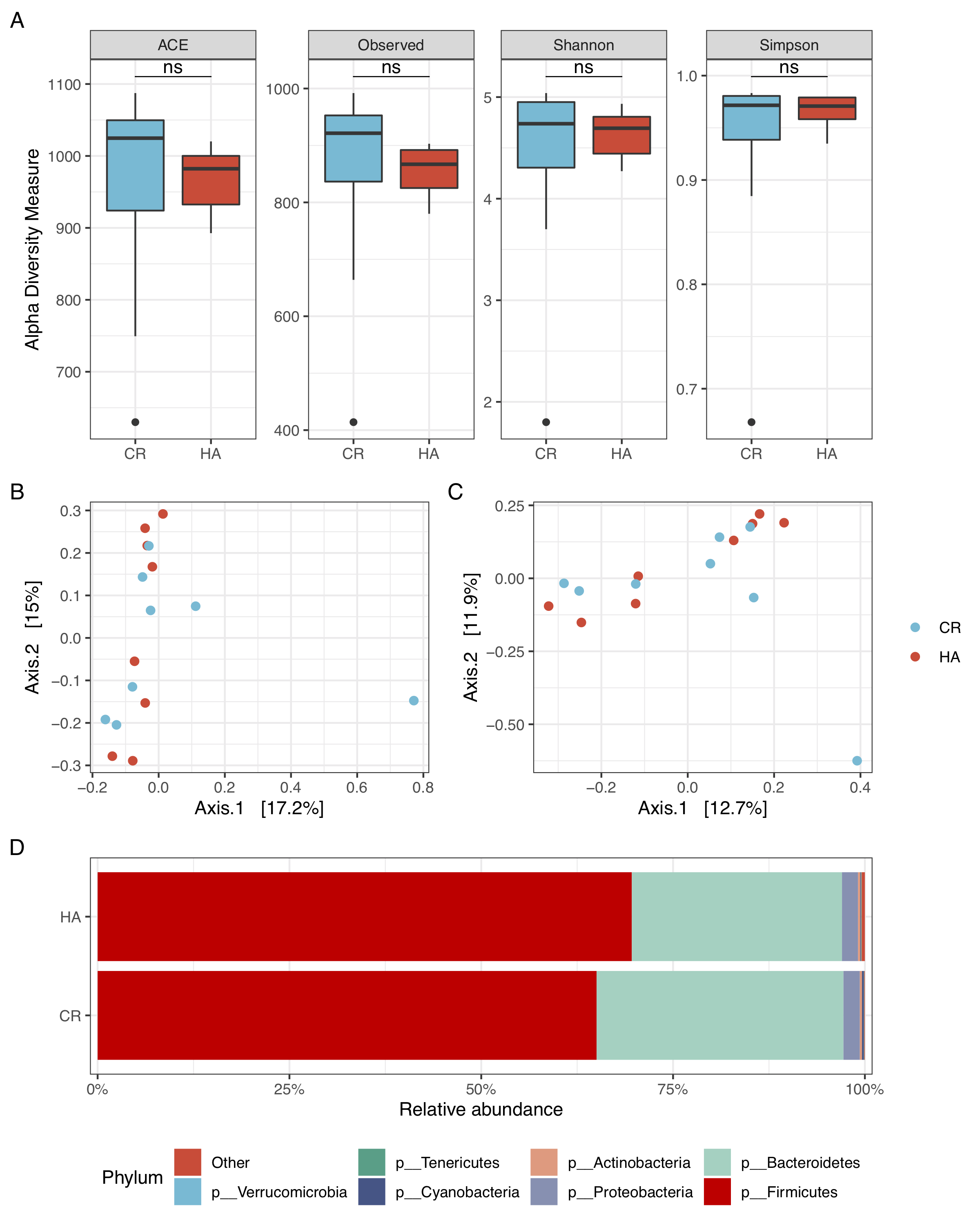

Supplement: Supplementary file 4 — Fig. S4. The degrees of OTUs in the four significant different genera of inferred ecological networks. P values are from Wilcoxon rank sum test. P value: *P < 0.05; ns, no significance P > 0.05. [file MBT2-15-276-s002.tiff]

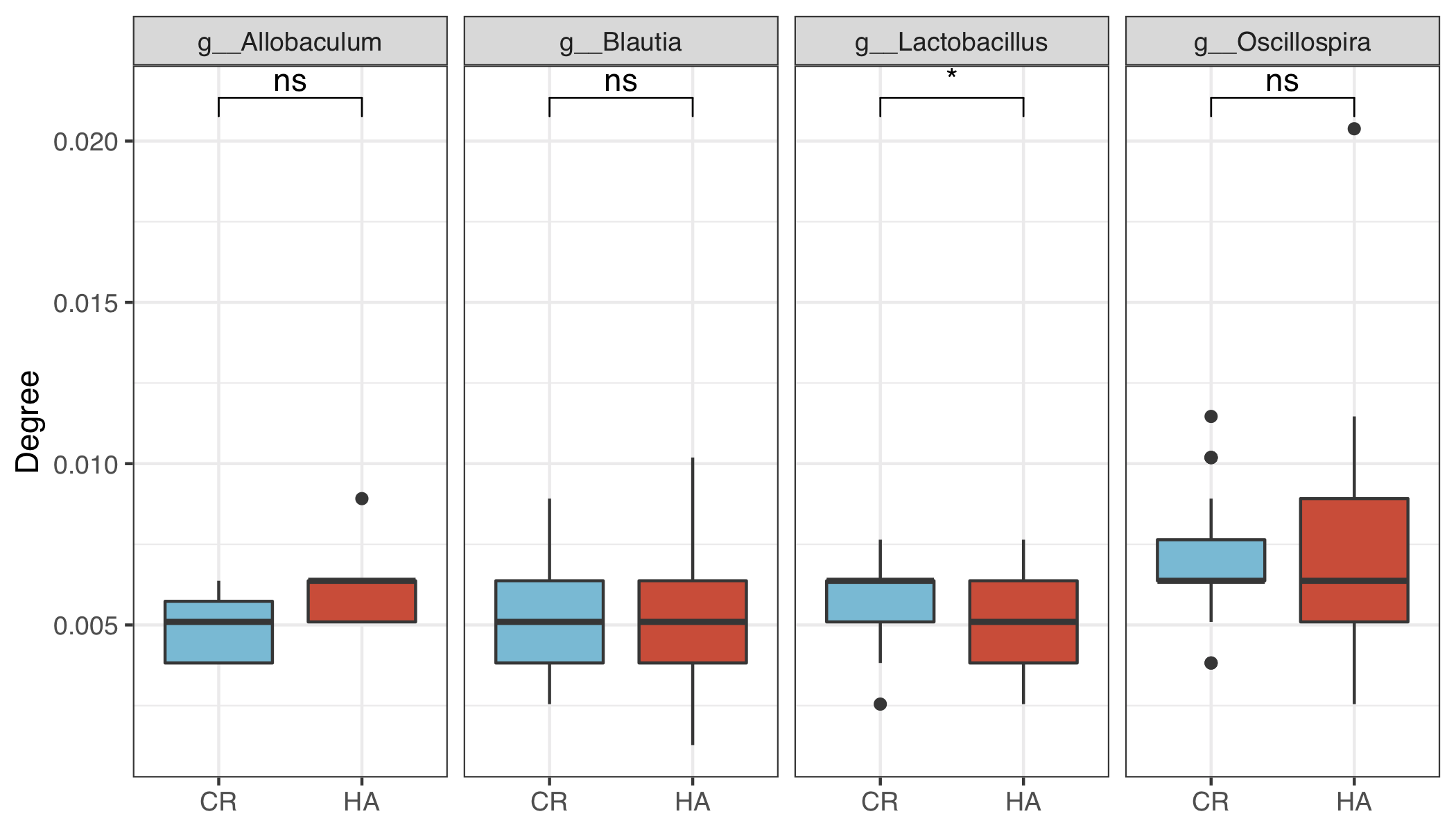

Supplement: Supplementary file 5 — Fig. S5. Targeted metabolomics profiling of celastrol. P values are from Wilcoxon rank sum test. P value: *P < 0.05. [file MBT2-15-276-s005.tiff]

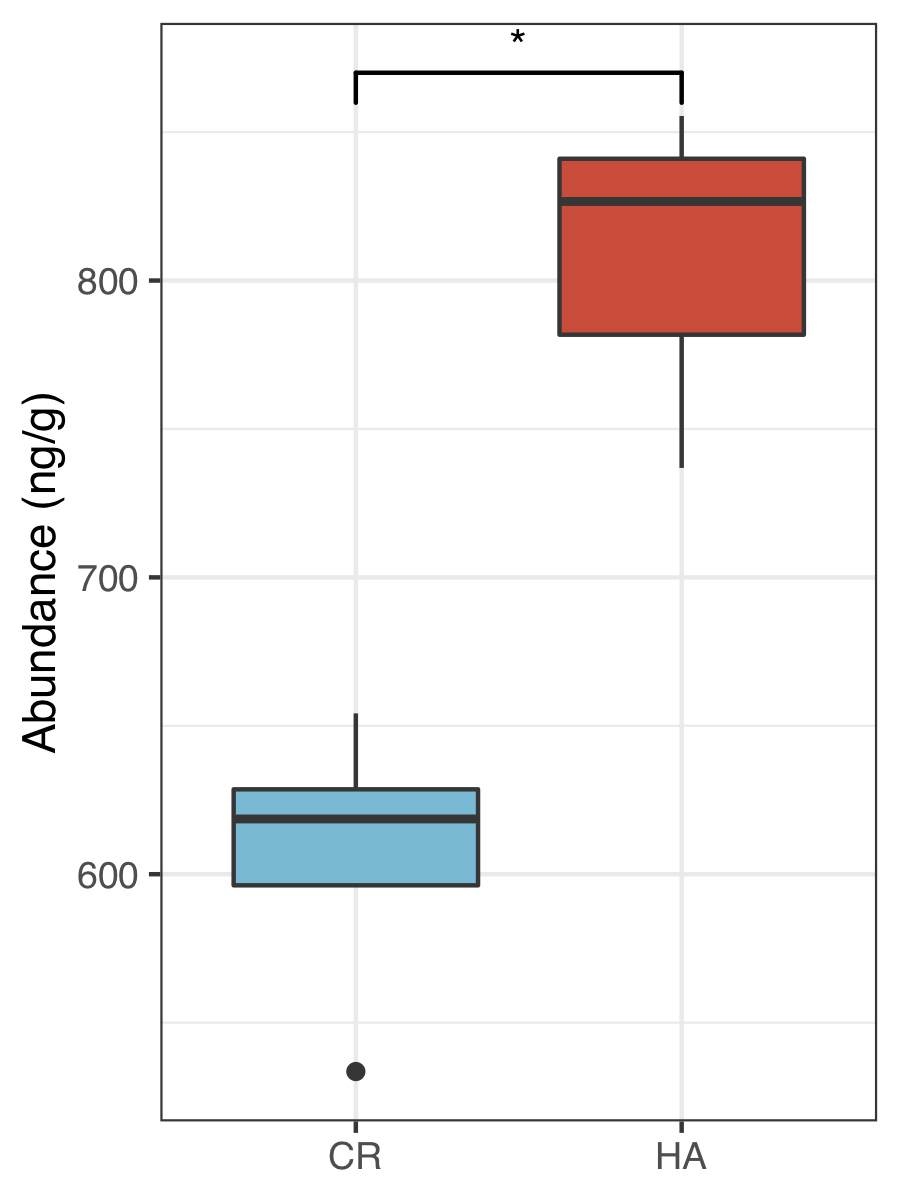

Supplement: Supplementary file 6 — Fig. S6. Diversity analysis on day 14. (A) Alpha diversity assessed by richness (ACE, Observed) and diversity (Shannon, Simpson). Boxes represent the interquartile ranges, and the inside black plots represent the median and circles are outliers. P values are from Wilcoxon rank sum test. Beta diversity assessed by principal coordinate analysis (PCoA) based on the Bray‐Curtis (B) and Jaccard (C) distances. Significant P‐values of Anosim and multi‐response permutation procedure (MRPP) between groups emphasize the differences in microbial community structure. (D) Relative abundance of bacterial phyla. P values: ng, no significance P > 0.05. [file MBT2-15-276-s007.tiff]
