# Supplementary material for: Alterations in the gut microbiome and metabolic profile in rats acclimated to high environmental temperature
Source: Microb Biotechnol. 2021 Feb 23;15(1):276–88. doi: 10.1111/1751-7915.13772 (PMC8719808; doi:10.1111/1751-7915.13772)
Supplement: Supplementary file 7 — Table S1. Wilcoxon rank‐sum test comparison of bacterial relative abundances at the phylum level. Table S2. Wilcoxon rank‐sum test comparison of bacterial relative abundances at the genus level. Table S3. Significantly different OTUs between HA and CR subjects which identified using DESeq2. Table S4. Differentially abundant taxa between HA and CR subjects which identified using LEfSe. Table S5. Significantly changed pathways after HA. Pathway activity analyses were predicted with both mummichog and GSEA methods using MetaboAnalystR. [file MBT2-15-276-s004.docx]

**Alterations in the gut microbiome and metabolic profile in rats acclimated to high environmental temperature**

Yang Cao^1✝^, Ying Liu^1✝^, Qingyang Dong^1✝^, Tao Wang^1^, Chao Niu^1*^

^1^Department of Environmental Medicine, Institute of Environmental and Operational Medicine, Tianjin 300050, China

***Correspondence:**

Chao Niu, niuchao601@126.com

^✝^These authors have contributed equally to this work.

# Supplementary tables

**Table S1.** Wilcoxon rank-sum test comparison of bacterial relative abundances at the phylum level.

| **Phylum** | **p-value** | **FDR** | **Mean relative abundance** | |
| --- | --- | --- | --- | --- |
|  |  |  | **CR** | **HA** |
| ***Unknown*** | 0.0104 | 0.1041 | 0.0009 | 0.0039 |
| ***p__Cyanobacteria*** | 0.083 | 0.4149 | 0.0025 | 0.0010 |
| ***p__Actinobacteria*** | 0.3823 | 0.7219 | 0.0026 | 0.0029 |
| ***p__Bacteroidetes*** | 0.5054 | 0.7219 | 0.3223 | 0.2736 |
| ***p__Firmicutes*** | 0.3823 | 0.7219 | 0.6497 | 0.6967 |
| ***p__Tenericutes*** | 0.5054 | 0.7219 | 0.0005 | 0.0006 |
| ***p__Verrucomicrobia*** | 0.3816 | 0.7219 | 0 | 0 |
| ***p__Fusobacteria*** | 0.5897 | 0.7372 | 0 | 0.0003 |
| ***p__Crenarchaeota*** | 0.7001 | 0.7779 | 0 | 0.0001 |
| ***p__Proteobacteria*** | 0.9591 | 0.9591 | 0.0214 | 0.0208 |

**Table S2.** Wilcoxon rank-sum test comparison of bacterial relative abundances at the genus level.

| ***Genus*** | **p-value** | **FDR** | **Mean relative abundance** | |
| --- | --- | --- | --- | --- |
|  |  |  | **CR** | **HA** |
| ***g__Blautia*** | 0.0006 | 0.0168 | 0.2593 | 0.0351 |
| ***g__Oscillospira*** | 0.0006 | 0.0168 | 0.0168 | 0.0638 |
| ***g__Lactobacillus*** | 0.003 | 0.0531 | 0.0233 | 0.0963 |
| ***g__Allobaculum*** | 0.007 | 0.0944 | 0.0051 | 0.0011 |
| ***g__Roseburia*** | 0.0148 | 0.1594 | 0.0147 | 0.0644 |
| ***g__Desulfovibrio*** | 0.0207 | 0.186 | 0.0021 | 0.0064 |
| ***g__Vagococcus*** | 0.0298 | 0.2302 | 0 | 0 |
| ***g__[Prevotella]*** | 0.0499 | 0.3367 | 0.043 | 0.0157 |
| ***g__Dehalobacterium*** | 0.0764 | 0.4481 | 0 | 0.0001 |
| ***g__Oxalobacter*** | 0.083 | 0.4481 | 0.0002 | 0.0003 |
| ***g__[Ruminococcus]*** | 0.1304 | 0.5416 | 0.0049 | 0.0081 |
| ***g__CF231*** | 0.1304 | 0.5416 | 0.013 | 0.0307 |
| ***g__rc4-4*** | 0.1304 | 0.5416 | 0.0017 | 0.005 |
| ***g__*** | 0.1903 | 0.5538 | 0.0103 | 0.0179 |
| ***g__Coprococcus*** | 0.1949 | 0.5538 | 0.0034 | 0.0079 |
| ***g__Dorea*** | 0.1949 | 0.5538 | 0.0032 | 0.0032 |
| ***g__Holdemania*** | 0.1949 | 0.5538 | 0.0008 | 0.0002 |
| ***g__Prevotella*** | 0.1605 | 0.5538 | 0.1985 | 0.1081 |
| ***g__Sutterella*** | 0.1605 | 0.5538 | 0.0177 | 0.011 |
| ***g__Psychrobacter*** | 0.262 | 0.7073 | 0 | 0 |
| ***g__Adlercreutzia*** | 0.4005 | 0.7723 | 0.0001 | 0.0001 |
| ***g__Akkermansia*** | 0.3816 | 0.7723 | 0 | 0 |
| ***g__Collinsella*** | 0.3823 | 0.7723 | 0.0012 | 0.0007 |
| ***g__Exiguobacterium*** | 0.3816 | 0.7723 | 0 | 0 |
| ***g__Lachnospira*** | 0.4002 | 0.7723 | 0 | 0.0013 |
| ***g__Ruminococcus*** | 0.3282 | 0.7723 | 0.0224 | 0.0376 |
| ***g__Sporosarcina*** | 0.3816 | 0.7723 | 0 | 0 |
| ***g__YRC22*** | 0.3823 | 0.7723 | 0.0016 | 0.0004 |
| ***g__Phascolarctobacterium*** | 0.4418 | 0.8227 | 0.0128 | 0.0053 |
| ***g__Lactococcus*** | 0.5069 | 0.8829 | 0 | 0 |
| ***g__Mycoplasma*** | 0.4936 | 0.8829 | 0.0004 | 0.0006 |
| ***g__[Eubacterium]*** | 0.5256 | 0.8847 | 0.0003 | 0.0002 |
| ***g__Bifidobacterium*** | 0.6854 | 0.8847 | 0 | 0 |
| ***g__Candidatus Arthromitus*** | 0.7209 | 0.8847 | 0.0009 | 0.0007 |
| ***g__Cetobacterium*** | 0.5897 | 0.8847 | 0 | 0.0003 |
| ***g__Clostridium*** | 0.5992 | 0.8847 | 0.0051 | 0.0066 |
| ***g__Erwinia*** | 0.5871 | 0.8847 | 0 | 0 |
| ***g__Moraxella*** | 0.7173 | 0.8847 | 0 | 0.0001 |
| ***g__Nitrosopumilus*** | 0.7001 | 0.8847 | 0 | 0.0001 |
| ***g__Sphingomonas*** | 0.6293 | 0.8847 | 0 | 0 |
| ***g__Stenotrophomonas*** | 0.7059 | 0.8847 | 0.0001 | 0 |
| ***g__Streptococcus*** | 0.6454 | 0.8847 | 0.001 | 0.0006 |
| ***g__Turicibacter*** | 0.6355 | 0.8847 | 0.0002 | 0.0001 |
| ***Unknown*** | 0.5737 | 0.8847 | 0.0252 | 0.0131 |
| ***g__5-7N15*** | 0.7984 | 0.8983 | 0.0035 | 0.0038 |
| ***g__Bacteroides*** | 0.7984 | 0.8983 | 0.0098 | 0.0095 |
| ***g__Megamonas*** | 0.7984 | 0.8983 | 0.0576 | 0.0302 |
| ***g__Rothia*** | 0.7984 | 0.8983 | 0.0001 | 0.0001 |
| ***g__Anaerostipes*** | 0.8481 | 0.916 | 0 | 0 |
| ***g__Haemophilus*** | 0.836 | 0.916 | 0 | 0.0001 |
| ***g__Coprobacillus*** | 0.8747 | 0.9262 | 0.0004 | 0.0008 |
| ***g__Butyricimonas*** | 0.958 | 0.9591 | 0.0005 | 0.0002 |
| ***g__p-75-a5*** | 0.9581 | 0.9591 | 0.0003 | 0.0002 |
| ***g__Parabacteroides*** | 0.9591 | 0.9591 | 0.0024 | 0.0011 |

**Table S3.** Significantly different OTUs between HA and CR subjects which identified using DESeq2.

| **OTU** | **Genus** | **baseMean** | **log2Fold**  **Change** | ***P*-value** | **Adjust *P*** |
| --- | --- | --- | --- | --- | --- |
| 849535 | ***g__Prevotella*** | 100.5145 | -8.2762 | 3.7315E-17 | 3.9106E-14 |
| 4474380 | ***g__Blautia*** | 1117.2239 | -7.1697 | 2.6358E-14 | 1.3812E-11 |
| 2774254 | ***g__*** | 554.9265 | -7.54 | 2.1849E-10 | 7.6325E-08 |
| 4412309 | ***g__Blautia*** | 32.4288 | -5.8402 | 1.6256E-09 | 3.7652E-07 |
| 318760 | ***g__Blautia*** | 93.6814 | -9.2869 | 1.7964E-09 | 3.7652E-07 |
| 2740950 | ***g__*** | 561.994 | -5.6986 | 9.4255E-08 | 1.6463E-05 |
| 22371 | ***g__*** | 75.4337 | 6.8934 | 1.5559E-07 | 2.3294E-05 |
| 827592 | ***g__Ruminococcus*** | 37.4421 | 3.6972 | 2.3664E-07 | 3.1000E-05 |
| 1111512 | ***g__*** | 59.4388 | 6.05 | 3.1491E-07 | 3.6670E-05 |
| 180999 | ***g__*** | 18.1004 | -5.3066 | 3.7795E-07 | 3.9609E-05 |
| 176312 | ***g__*** | 115.1398 | -5.0027 | 7.2020E-07 | 6.8616E-05 |
| 4407278 | ***g__Blautia*** | 9.1901 | -5.505 | 7.9296E-07 | 6.9252E-05 |
| 178859 | ***g__Ruminococcus*** | 42.5266 | 3.8429 | 1.2004E-06 | 9.6767E-05 |
| 131571 | ***g__Blautia*** | 9.2299 | -4.5134 | 1.4759E-06 | 1.0312E-04 |
| 4296216 | ***g__Ruminococcus*** | 254.6503 | -5.9914 | 1.4212E-06 | 1.0312E-04 |
| 230268 | ***g__Ruminococcus*** | 19.4252 | 5.8021 | 1.6323E-06 | 1.0692E-04 |
| New.ReferenceOTU84 | ***g__*** | 19.5017 | -8.1121 | 1.9300E-06 | 1.1898E-04 |
| 135956 | ***g__Lactobacillus*** | 1139.0253 | 3.174 | 2.7521E-06 | 1.6023E-04 |
| 278123 | ***g__*** | 106.2636 | 5.3508 | 5.7688E-06 | 3.1820E-04 |
| 4460560 | ***g__Ruminococcus*** | 8.6972 | 6.1841 | 7.6333E-06 | 3.8068E-04 |
| 203700 | ***g__*** | 35.0684 | 4.0225 | 7.3461E-06 | 3.8068E-04 |
| 68844 | ***g__*** | 29.9647 | 6.8747 | 7.9913E-06 | 3.8068E-04 |
| 181675 | ***g__Blautia*** | 11.4101 | -3.8931 | 9.5660E-06 | 4.1625E-04 |
| New.ReferenceOTU41653 | ***g__[Prevotella]*** | 82.2922 | -5.0368 | 9.3306E-06 | 4.1625E-04 |
| New.ReferenceOTU66045 | ***g__*** | 21.3665 | 5.5448 | 9.9295E-06 | 4.1625E-04 |
| 4440360 | ***g__*** | 38.1794 | 5.9539 | 1.0980E-05 | 4.4256E-04 |
| 1106830 | ***g__Coprococcus*** | 26.2108 | 4.108 | 1.1848E-05 | 4.5988E-04 |
| 164664 | ***g__Lactobacillus*** | 61.1278 | 3.0019 | 1.4285E-05 | 5.1622E-04 |
| 229958 | ***g__Oscillospira*** | 9.004 | 6.2349 | 1.4229E-05 | 5.1622E-04 |
| New.ReferenceOTU685 | ***g__*** | 19.6517 | 6.2595 | 1.5475E-05 | 5.4060E-04 |
| 181985 | ***g__Roseburia*** | 93.0104 | 6.547 | 1.7327E-05 | 5.7342E-04 |
| 215266 | ***g__Clostridium*** | 13.0138 | -5.2479 | 1.7509E-05 | 5.7342E-04 |
| 4420417 | ***g__*** | 76.3787 | 2.068 | 1.9322E-05 | 6.1361E-04 |
| New.ReferenceOTU42502 | ***g__[Prevotella]*** | 25.3588 | -5.3145 | 2.1675E-05 | 6.4901E-04 |
| 4391009 | ***g__Sutterella*** | 28.212 | -6.8339 | 2.1451E-05 | 6.4901E-04 |
| 269125 | ***g__Lactobacillus*** | 25.3302 | 3.4342 | 2.6025E-05 | 7.5763E-04 |
| 2120775 | ***g__*** | 18.3014 | 4.5384 | 3.1662E-05 | 8.9680E-04 |
| New.ReferenceOTU79 | ***g__*** | 43.3376 | 4.4659 | 3.4413E-05 | 9.2474E-04 |
| 3998909 | ***g__*** | 272.6271 | 3.8238 | 3.4119E-05 | 9.2474E-04 |
| 158297 | ***g__Ruminococcus*** | 9.3153 | 4.2788 | 3.5936E-05 | 9.4153E-04 |
| 4459184 | ***g__*** | 57.831 | 4.415 | 4.2811E-05 | 1.0682E-03 |
| 10512 | ***g__Desulfovibrio*** | 145.7396 | 2.904 | 4.1991E-05 | 1.0682E-03 |
| 2235671 | ***g__Blautia*** | 16.4927 | -6.0765 | 4.4858E-05 | 1.0933E-03 |
| 2366688 | ***g__*** | 271.8655 | 3.1882 | 4.6928E-05 | 1.0993E-03 |
| 4430497 | ***g__*** | 67.3006 | 5.4324 | 4.7204E-05 | 1.0993E-03 |
| 95910 | ***g__*** | 14.1732 | 6.1492 | 5.8298E-05 | 1.3158E-03 |
| New.ReferenceOTU91 | ***g__*** | 4.5138 | 5.2404 | 5.9008E-05 | 1.3158E-03 |
| 174516 | ***g__Clostridium*** | 126.7947 | -4.3004 | 6.1783E-05 | 1.3489E-03 |
| New.ReferenceOTU46938 | ***g__*** | 6.1884 | 4.2318 | 6.7967E-05 | 1.4537E-03 |
| 795046 | ***g__*** | 20.5793 | 4.939 | 6.9516E-05 | 1.4571E-03 |
| 295023 | ***g__Blautia*** | 6.1094 | -4.4107 | 7.2233E-05 | 1.4843E-03 |
| 4326861 | ***g__*** | 9.5807 | -4.8585 | 7.7322E-05 | 1.5583E-03 |
| New.ReferenceOTU65951 | ***g__*** | 9.0214 | 5.4931 | 8.0766E-05 | 1.5970E-03 |
| 279340 | ***g__*** | 7.0858 | 5.1401 | 8.2483E-05 | 1.6008E-03 |
| 4423384 | ***g__Blautia*** | 73.2177 | -4.3694 | 9.1841E-05 | 1.7500E-03 |
| 193446 | ***g__Blautia*** | 12.4017 | -6.3559 | 1.0166E-04 | 1.8368E-03 |
| 185836 | ***g__Blautia*** | 15.4673 | -4.0008 | 1.0135E-04 | 1.8368E-03 |
| New.ReferenceOTU7297 | ***g__*** | 18.3593 | 4.2108 | 1.0073E-04 | 1.8368E-03 |
| New.ReferenceOTU87 | ***g__*** | 15.6849 | 4.1934 | 1.2014E-04 | 2.1340E-03 |
| 180629 | ***g__Blautia*** | 57.7995 | -3.8807 | 1.2913E-04 | 2.2185E-03 |
| 189624 | ***g__*** | 12.7027 | -5.1856 | 1.2909E-04 | 2.2185E-03 |
| 4429536 | ***g__Blautia*** | 11.3972 | -5.5665 | 1.3738E-04 | 2.3222E-03 |
| 383992 | ***g__*** | 45.6644 | 3.1351 | 1.6058E-04 | 2.6713E-03 |
| 175685 | ***g__Blautia*** | 23.4136 | -3.5735 | 1.7647E-04 | 2.8896E-03 |
| New.ReferenceOTU117 | ***g__*** | 7.306 | 4.8097 | 1.8699E-04 | 3.0149E-03 |
| 4310326 | ***g__Allobaculum*** | 102.3869 | -3.0019 | 2.0988E-04 | 3.3327E-03 |
| 231169 | ***g__*** | 6.5766 | 3.5367 | 2.3502E-04 | 3.6761E-03 |
| 4447176 | ***g__*** | 21.3886 | 2.5113 | 2.4501E-04 | 3.6873E-03 |
| 4456702 | ***g__Ruminococcus*** | 6.9203 | 3.7621 | 2.4629E-04 | 3.6873E-03 |
| New.ReferenceOTU9686 | ***g__*** | 7.6977 | 6.0085 | 2.3931E-04 | 3.6873E-03 |
| 4402077 | ***g__*** | 17.2522 | 5.6713 | 2.9770E-04 | 4.3942E-03 |
| 4468466 | ***g__*** | 63.9686 | -5.2932 | 3.0771E-04 | 4.4789E-03 |
| 4380191 | ***g__Roseburia*** | 22.9338 | 4.062 | 3.1986E-04 | 4.5313E-03 |
| New.ReferenceOTU9440 | ***g__Lactobacillus*** | 7.5083 | 2.8008 | 3.1996E-04 | 4.5313E-03 |
| New.ReferenceOTU5162 | ***g__Allobaculum*** | 11.5215 | -3.4825 | 3.2828E-04 | 4.5871E-03 |
| 4430499 | ***g__*** | 13.3257 | 4.2449 | 3.5085E-04 | 4.8381E-03 |
| 174667 | ***g__*** | 7.2967 | 4.4635 | 3.5972E-04 | 4.8959E-03 |
| New.ReferenceOTU9419 | ***g__Ruminococcus*** | 7.2415 | 4.473 | 3.6675E-04 | 4.9276E-03 |
| 4481359 | ***g__*** | 29.1665 | 3.375 | 3.8268E-04 | 5.0766E-03 |
| 336375 | ***g__Blautia*** | 238.692 | -3.3671 | 3.9056E-04 | 5.1163E-03 |
| 179549 | ***g__Blautia*** | 639.0894 | -3.3864 | 4.2459E-04 | 5.4935E-03 |
| New.ReferenceOTU61266 | ***g__Phascolarctobacterium*** | 6.2203 | -2.8817 | 4.3667E-04 | 5.5692E-03 |
| 258725 | ***g__Oscillospira*** | 18.5397 | 6.5427 | 4.4336E-04 | 5.5692E-03 |
| 3529330 | ***g__*** | 10.4371 | 4.4247 | 4.4639E-04 | 5.5692E-03 |
| 172640 | ***g__*** | 16.243 | 3.8076 | 4.8843E-04 | 5.8836E-03 |
| 199336 | ***g__Coprococcus*** | 45.6296 | -4.7012 | 4.8205E-04 | 5.8836E-03 |
| 4396297 | ***g__*** | 16.3539 | -4.1092 | 4.8513E-04 | 5.8836E-03 |
| 169428 | ***g__Lactobacillus*** | 48.8008 | 2.7037 | 5.0253E-04 | 5.9846E-03 |
| 635495 | ***g__*** | 6.6394 | -5.4342 | 5.2549E-04 | 6.1878E-03 |
| 443853 | ***g__*** | 3.0849 | 3.9098 | 5.9121E-04 | 6.8843E-03 |
| 4465907 | ***g__Blautia*** | 8312.8874 | -3.4606 | 6.0004E-04 | 6.9104E-03 |
| 181330 | ***g__Blautia*** | 181.5245 | -3.5126 | 6.0730E-04 | 6.9179E-03 |
| 4429301 | ***g__Blautia*** | 200.1245 | -3.5213 | 6.1890E-04 | 6.9743E-03 |
| New.ReferenceOTU7710 | ***g__Phascolarctobacterium*** | 82.8242 | -2.6081 | 6.3317E-04 | 7.0592E-03 |
| New.ReferenceOTU140 | ***g__*** | 3.7767 | -5.7357 | 6.4552E-04 | 7.1211E-03 |
| New.ReferenceOTU62900 | ***g__*** | 27.1184 | 3.7501 | 6.9079E-04 | 7.5411E-03 |
| 4296496 | ***g__*** | 6.1358 | 4.9268 | 7.2070E-04 | 7.6046E-03 |
| 191098 | ***g__*** | 29.5976 | 2.6183 | 7.2216E-04 | 7.6046E-03 |
| 261590 | ***g__*** | 8.9975 | -3.6811 | 7.2173E-04 | 7.6046E-03 |
| 845272 | ***g__Oscillospira*** | 14.8045 | 2.3453 | 7.3289E-04 | 7.6046E-03 |
| New.ReferenceOTU66374 | ***g__*** | 12.4605 | 4.3471 | 7.2878E-04 | 7.6046E-03 |
| 4409730 | ***NA*** | 863.5467 | -2.7556 | 7.4315E-04 | 7.6355E-03 |
| New.ReferenceOTU32 | ***g__*** | 4.9308 | 4.9811 | 7.9986E-04 | 8.1384E-03 |
| 278609 | ***g__Oscillospira*** | 5.9163 | 5.2539 | 8.1541E-04 | 8.2168E-03 |
| 199067 | ***g__Coprococcus*** | 36.4585 | 2.8367 | 8.8531E-04 | 8.6711E-03 |
| 4417539 | ***g__Ruminococcus*** | 8.9569 | 4.3047 | 8.7243E-04 | 8.6711E-03 |
| 185610 | ***g__*** | 164.0059 | -3.2614 | 8.7765E-04 | 8.6711E-03 |
| New.ReferenceOTU9345 | ***g__*** | 5.0392 | 3.3194 | 8.9506E-04 | 8.6854E-03 |
| 185240 | ***g__Blautia*** | 15.0923 | -4.0597 | 9.1350E-04 | 8.7031E-03 |
| 185969 | ***g__Blautia*** | 52.2498 | -3.7271 | 9.0623E-04 | 8.7031E-03 |
| 278675 | ***g__*** | 10.0724 | -2.206 | 9.7989E-04 | 9.2516E-03 |
| 4435236 | ***g__Blautia*** | 4.2345 | -2.8026 | 9.9841E-04 | 9.2596E-03 |
| 197600 | ***g__*** | 38.8158 | 3.3829 | 9.9216E-04 | 9.2596E-03 |
| 227565 | ***g__*** | 9.7 | -2.8603 | 1.0092E-03 | 9.2772E-03 |
| New.ReferenceOTU49 | ***g__*** | 25.5113 | 2.4264 | 1.0239E-03 | 9.3306E-03 |
| New.ReferenceOTU37174 | ***g__*** | 4.0321 | -5.4572 | 1.0440E-03 | 9.4322E-03 |
| 4311621 | ***g__Phascolarctobacterium*** | 161.4896 | -2.1888 | 1.0538E-03 | 9.4395E-03 |
| 4359832 | ***g__*** | 5.4043 | 5.1203 | 1.0873E-03 | 9.5171E-03 |
| 190676 | ***g__Oscillospira*** | 7.986 | 2.5739 | 1.0897E-03 | 9.5171E-03 |
| 279306 | ***g__*** | 9.5966 | 4.5806 | 1.0795E-03 | 9.5171E-03 |
| 3750380 | ***g__*** | 6.0301 | 4.51 | 1.1153E-03 | 9.6601E-03 |
| 301765 | ***g__*** | 22.4636 | 4.6909 | 1.1347E-03 | 9.6679E-03 |
| New.ReferenceOTU64928 | ***g__Allobaculum*** | 15.2492 | -2.8409 | 1.1263E-03 | 9.6679E-03 |
| 110192 | ***g__Oscillospira*** | 7.9857 | 4.9441 | 1.1722E-03 | 9.8481E-03 |
| 383659 | ***g__*** | 6.0783 | 4.1126 | 1.1746E-03 | 9.8481E-03 |
| 390497 | ***g__Oscillospira*** | 15.63 | 3.8988 | 1.1929E-03 | 9.9217E-03 |
| 4472130 | ***g__*** | 54.1067 | 3.4474 | 1.2232E-03 | 1.0094E-02 |
| New.ReferenceOTU8882 | ***g__*** | 21.8235 | -5.0114 | 1.2466E-03 | 1.0207E-02 |
| 365341 | ***g__Blautia*** | 49.2733 | -3.4604 | 1.2917E-03 | 1.0494E-02 |
| 1107461 | ***g__*** | 19.5855 | 3.0951 | 1.3697E-03 | 1.0773E-02 |
| 1109297 | ***g__Dorea*** | 8.6223 | 3.5217 | 1.3703E-03 | 1.0773E-02 |
| 70217 | ***g__Prevotella*** | 4.9047 | 5.3549 | 1.4084E-03 | 1.0773E-02 |
| 187264 | ***g__*** | 144.057 | -2.816 | 1.3717E-03 | 1.0773E-02 |
| New.ReferenceOTU86 | ***g__*** | 5.2388 | 5.4515 | 1.3986E-03 | 1.0773E-02 |
| 450755 | ***g__*** | 2.9783 | 4.6484 | 1.3797E-03 | 1.0773E-02 |
| New.ReferenceOTU65724 | ***g__*** | 4.2762 | 4.3971 | 1.3910E-03 | 1.0773E-02 |
| 179744 | ***g__*** | 12.6489 | -4.6844 | 1.3464E-03 | 1.0773E-02 |
| 181844 | ***g__Blautia*** | 17.3173 | -4.1198 | 1.4723E-03 | 1.1100E-02 |
| 291315 | ***g__Phascolarctobacterium*** | 42.1886 | -2.6243 | 1.4640E-03 | 1.1100E-02 |
| 68845 | ***g__Blautia*** | 36.7873 | -3.5591 | 1.5737E-03 | 1.1780E-02 |
| 183857 | ***g__Blautia*** | 20.7158 | -3.2802 | 1.6037E-03 | 1.1920E-02 |
| 4296217 | ***g__Blautia*** | 728.859 | -3.2777 | 1.6852E-03 | 1.2180E-02 |
| 4359984 | ***g__*** | 10.3109 | 3.898 | 1.6756E-03 | 1.2180E-02 |
| 193279 | ***g__Adlercreutzia*** | 3.6219 | -3.5881 | 1.6651E-03 | 1.2180E-02 |
| New.ReferenceOTU7711 | ***g__Allobaculum*** | 17.49 | -2.5236 | 1.6571E-03 | 1.2180E-02 |
| 1106683 | ***g__*** | 5.8677 | 4.8533 | 1.7333E-03 | 1.2357E-02 |
| 4327763 | ***g__*** | 49.8089 | -4.1086 | 1.7289E-03 | 1.2357E-02 |
| New.ReferenceOTU120 | ***g__Collinsella*** | 13.2329 | -2.8763 | 1.7776E-03 | 1.2587E-02 |
| 173916 | ***g__*** | 5.8937 | 4.1643 | 1.8153E-03 | 1.2768E-02 |
| 2256427 | ***g__Blautia*** | 11.3337 | -3.2737 | 1.8735E-03 | 1.3090E-02 |
| 4435934 | ***g__*** | 3.7019 | 3.7965 | 2.0191E-03 | 1.4014E-02 |
| 4416570 | ***g__*** | 471.3563 | -3.1233 | 2.0535E-03 | 1.4158E-02 |
| 164811 | ***g__*** | 3.5971 | 4.5095 | 2.0906E-03 | 1.4320E-02 |
| 4459196 | ***g__Lachnospira*** | 9.4662 | 4.8945 | 2.1236E-03 | 1.4452E-02 |
| 199491 | ***g__*** | 28.7296 | -4.3147 | 2.1451E-03 | 1.4503E-02 |
| 187207 | ***g__Blautia*** | 60.6999 | -3.4889 | 2.1667E-03 | 1.4556E-02 |
| 1109178 | ***g__[Ruminococcus]*** | 18.011 | 3.9338 | 2.2897E-03 | 1.5199E-02 |
| 2801994 | ***g__Phascolarctobacterium*** | 95.6966 | -2.1778 | 2.2971E-03 | 1.5199E-02 |
| 168541 | ***g__*** | 7.5225 | 3.9421 | 2.3059E-03 | 1.5199E-02 |
| New.ReferenceOTU9350 | ***g__Phascolarctobacterium*** | 37.7826 | -2.3877 | 2.3383E-03 | 1.5316E-02 |
| 292444 | ***g__YRC22*** | 62.0621 | -2.5302 | 2.3762E-03 | 1.5468E-02 |
| 4296701 | ***g__Oscillospira*** | 5.4132 | 2.441 | 2.4357E-03 | 1.5756E-02 |
| New.ReferenceOTU62439 | ***g__*** | 2.0182 | 4.0936 | 2.4506E-03 | 1.5756E-02 |
| 784256 | ***g__*** | 6.6555 | 2.0543 | 2.4938E-03 | 1.5936E-02 |
| 318162 | ***g__*** | 11.933 | 3.2642 | 2.5186E-03 | 1.5997E-02 |
| 4383135 | ***g__*** | 9.0407 | 4.1232 | 2.5609E-03 | 1.6168E-02 |
| New.ReferenceOTU37755 | ***g__Allobaculum*** | 16.9809 | -2.7063 | 2.5924E-03 | 1.6269E-02 |
| 172859 | ***g__*** | 13.0448 | 2.822 | 2.6248E-03 | 1.6374E-02 |
| New.ReferenceOTU47180 | ***g__*** | 2.8592 | 4.188 | 2.6986E-03 | 1.6735E-02 |
| 176298 | ***g__*** | 21.7379 | -3.0778 | 2.8273E-03 | 1.7430E-02 |
| 128300 | ***g__Lactobacillus*** | 3.2492 | -4.3995 | 2.8705E-03 | 1.7490E-02 |
| New.ReferenceOTU51166 | ***g__Phascolarctobacterium*** | 10.9386 | -2.2401 | 2.8612E-03 | 1.7490E-02 |
| New.ReferenceOTU65850 | ***g__Phascolarctobacterium*** | 23.8603 | -2.4631 | 2.8970E-03 | 1.7549E-02 |
| New.ReferenceOTU96 | ***g__*** | 18.6809 | 3.2293 | 2.9192E-03 | 1.7582E-02 |
| 1111745 | ***g__*** | 25.1996 | 3.251 | 2.9619E-03 | 1.7637E-02 |
| 4394350 | ***g__*** | 8.5354 | 3.0848 | 2.9574E-03 | 1.7637E-02 |
| 262944 | ***g__*** | 6.385 | 2.8785 | 3.0003E-03 | 1.7764E-02 |
| 291055 | ***g__Prevotella*** | 194.7181 | -2.1835 | 3.0701E-03 | 1.8076E-02 |
| 4462739 | ***g__Blautia*** | 29.4288 | -3.8994 | 3.0985E-03 | 1.8141E-02 |
| 205613 | ***g__Coprococcus*** | 4.9258 | 2.7309 | 3.2049E-03 | 1.8556E-02 |
| 1107586 | ***g__Coprococcus*** | 31.3251 | 2.5291 | 3.1877E-03 | 1.8556E-02 |
| New.ReferenceOTU167 | ***g__Allobaculum*** | 25.5735 | -2.252 | 3.3560E-03 | 1.9324E-02 |
| 174320 | ***g__*** | 3.6633 | 3.731 | 3.4428E-03 | 1.9716E-02 |
| 512494 | ***g__Phascolarctobacterium*** | 6.5935 | -2.9538 | 3.5536E-03 | 2.0240E-02 |
| 169234 | ***g__*** | 2.8258 | 3.3862 | 3.6031E-03 | 2.0411E-02 |
| 105109 | ***g__*** | 8.1208 | -4.1304 | 3.8343E-03 | 2.1604E-02 |
| 310499 | ***g__*** | 5.8414 | 3.8415 | 3.8586E-03 | 2.1625E-02 |
| 169031 | ***g__Phascolarctobacterium*** | 13.0637 | -2.5679 | 3.9556E-03 | 2.1934E-02 |
| 337494 | ***g__*** | 2.8585 | 4.5832 | 3.9439E-03 | 2.1934E-02 |
| New.ReferenceOTU119 | ***g__*** | 8.5902 | 2.5538 | 4.1124E-03 | 2.2565E-02 |
| New.ReferenceOTU68900 | ***g__Phascolarctobacterium*** | 9.4104 | -2.0585 | 4.0970E-03 | 2.2565E-02 |
| 396239 | ***g__*** | 375.3289 | 2.4612 | 4.4340E-03 | 2.4022E-02 |
| 782984 | ***g__*** | 157.8316 | -4.0297 | 4.4200E-03 | 2.4022E-02 |
| 1105326 | ***g__*** | 5.5932 | 4.126 | 4.4469E-03 | 2.4022E-02 |
| 804770 | ***NA*** | 6.4058 | -3.0967 | 4.5551E-03 | 2.4481E-02 |
| 98048 | ***g__Oscillospira*** | 3.7536 | 4.9731 | 4.5806E-03 | 2.4492E-02 |
| 181335 | ***g__Blautia*** | 53.4452 | -3.3144 | 5.0376E-03 | 2.6664E-02 |
| New.ReferenceOTU9637 | ***g__*** | 4.2997 | 4.0104 | 5.0169E-03 | 2.6664E-02 |
| 4462867 | ***g__Blautia*** | 47.1734 | -3.592 | 5.5040E-03 | 2.8986E-02 |
| 4481613 | ***g__Collinsella*** | 5.3928 | -3.8623 | 5.5487E-03 | 2.9075E-02 |
| 839137 | ***g__*** | 5.9449 | 2.932 | 5.6244E-03 | 2.9325E-02 |
| New.ReferenceOTU48311 | ***g__*** | 92.056 | -1.8524 | 5.8825E-03 | 3.0519E-02 |
| New.ReferenceOTU59566 | ***g__*** | 18.3279 | 1.7016 | 5.9501E-03 | 3.0567E-02 |
| 542934 | ***g__*** | 7.6587 | -2.8487 | 5.9352E-03 | 3.0567E-02 |
| New.ReferenceOTU7426 | ***g__*** | 8.0672 | 2.7288 | 6.1060E-03 | 3.1215E-02 |
| New.ReferenceOTU5873 | ***g__*** | 2.8755 | 3.7708 | 6.2129E-03 | 3.1607E-02 |
| 423830 | ***g__*** | 23.3058 | 2.685 | 6.3879E-03 | 3.2341E-02 |
| 1109860 | ***g__Dorea*** | 7.7907 | 2.9928 | 6.5195E-03 | 3.2848E-02 |
| 4465124 | ***g__*** | 270.1454 | -3.3105 | 6.6105E-03 | 3.3106E-02 |
| New.ReferenceOTU101 | ***g__*** | 2.375 | 3.5124 | 6.6339E-03 | 3.3106E-02 |
| 258165 | ***g__*** | 2.7062 | 3.7016 | 6.6699E-03 | 3.3128E-02 |
| 320603 | ***g__*** | 34.9788 | -4.1889 | 6.8163E-03 | 3.3696E-02 |
| 4484034 | ***g__Bacteroides*** | 2.3068 | 3.4587 | 6.8793E-03 | 3.3847E-02 |
| 164242 | ***g__*** | 2.2372 | 3.0154 | 6.9117E-03 | 3.3848E-02 |
| New.ReferenceOTU9588 | ***g__*** | 3.5929 | 3.3957 | 7.1869E-03 | 3.5032E-02 |
| 18619 | ***g__*** | 17.2844 | 2.7268 | 7.2724E-03 | 3.5239E-02 |
| 262673 | ***g__*** | 30.5978 | -1.8159 | 7.2967E-03 | 3.5239E-02 |
| 4356342 | ***g__[Ruminococcus]*** | 48.1366 | 3.0952 | 7.4386E-03 | 3.5760E-02 |
| 291206 | ***g__*** | 4.9164 | 3.5785 | 7.5089E-03 | 3.5933E-02 |
| 805672 | ***g__Oscillospira*** | 10.8618 | 1.9387 | 7.6517E-03 | 3.6400E-02 |
| 1109929 | ***g__*** | 13.1538 | 2.3604 | 7.6759E-03 | 3.6400E-02 |
| 4309927 | ***g__*** | 9.7686 | 3.8949 | 8.1383E-03 | 3.8419E-02 |
| 301876 | ***NA*** | 5.768 | -4.0421 | 8.2007E-03 | 3.8540E-02 |
| 333426 | ***g__*** | 3.2902 | -3.7727 | 8.3932E-03 | 3.9268E-02 |
| 186022 | ***g__Blautia*** | 174.9336 | -2.8575 | 8.4359E-03 | 3.9292E-02 |
| 835154 | ***g__*** | 4.2653 | 2.616 | 8.6179E-03 | 3.9963E-02 |
| 171532 | ***g__*** | 5.0961 | 3.286 | 8.7253E-03 | 4.0282E-02 |
| 2119695 | ***g__*** | 14.8747 | -2.5587 | 8.9311E-03 | 4.1036E-02 |
| 1077336 | ***g__*** | 12.1855 | 2.4869 | 8.9669E-03 | 4.1036E-02 |
| 275237 | ***g__Phascolarctobacterium*** | 8.2674 | -2.5671 | 9.3253E-03 | 4.2491E-02 |
| 362854 | ***g__*** | 37.4014 | -3.0807 | 9.4086E-03 | 4.2685E-02 |
| 4446238 | ***g__Dorea*** | 9.8808 | -2.3773 | 9.8166E-03 | 4.3778E-02 |
| New.ReferenceOTU46 | ***g__*** | 3.91 | 2.873 | 9.7958E-03 | 4.3778E-02 |
| 278758 | ***g__Oscillospira*** | 2.255 | 3.8429 | 9.7070E-03 | 4.3778E-02 |
| 195445 | ***g__*** | 2.4491 | 3.1561 | 9.8095E-03 | 4.3778E-02 |
| New.ReferenceOTU61071 | ***g__*** | 3.3532 | 3.6448 | 1.0169E-02 | 4.5157E-02 |
| 374557 | ***g__*** | 104.179 | 2.7562 | 1.0461E-02 | 4.6258E-02 |
| New.ReferenceOTU6265 | ***g__Lactobacillus*** | 7.9625 | 2.6467 | 1.0662E-02 | 4.6804E-02 |
| 4468391 | ***g__Ruminococcus*** | 8.9105 | 3.1938 | 1.0674E-02 | 4.6804E-02 |
| 1129971 | ***g__*** | 4.0795 | -3.0405 | 1.0897E-02 | 4.7583E-02 |
| 3568710 | ***NA*** | 5.8018 | -2.9586 | 1.1132E-02 | 4.8408E-02 |
| New.ReferenceOTU4274 | ***g__Phascolarctobacterium*** | 9.7618 | -1.8539 | 1.1247E-02 | 4.8504E-02 |
| 4432234 | ***g__*** | 12.2484 | 3.5821 | 1.1203E-02 | 4.8504E-02 |
| 4464065 | ***g__*** | 12.3603 | 1.9247 | 1.1665E-02 | 4.9896E-02 |
| 15728 | ***g__Holdemania*** | 36.5956 | -2.5406 | 1.1665E-02 | 4.9896E-02 |
| New.ReferenceOTU42336 | ***g__[Prevotella]*** | 108.2097 | -2.7501 | 1.1781E-02 | 4.9985E-02 |
| 270571 | ***g__Oscillospira*** | 8.0582 | 2.7279 | 1.1736E-02 | 4.9985E-02 |

**Table S4.** Differentially abundant taxa between HA and CR subjects which identified using LEfSe.

| **Taxa** | **Enriched group** | **LDA score** | ***P*-value** |
| --- | --- | --- | --- |
| ***.f__Ruminococcaceae*** | HA | 3.9482 | 0.0274 |
| ***g__Lactobacillus*** | HA | 3.9292 | 0.0046 |
| ***f__Lactobacillaceae*** | HA | 3.9292 | 0.0046 |
| ***o__Lactobacillales*** | HA | 3.9272 | 0.0046 |
| ***c__Bacilli*** | HA | 3.9266 | 0.0046 |
| ***f__S24_7*** | HA | 3.7926 | 0.0033 |
| ***g__Oscillospira*** | HA | 3.7351 | 0.0016 |
| ***g__Roseburia*** | HA | 3.7205 | 0.0157 |
| ***g__Vagococcus*** | HA | 2.9128 | 0.0256 |
| ***o__Desulfovibrionales*** | HA | 2.8248 | 0.0274 |
| ***f__Desulfovibrionaceae*** | HA | 2.8248 | 0.0274 |
| ***c__Deltaproteobacteria*** | HA | 2.8248 | 0.0274 |
| ***g__Desulfovibrio*** | HA | 2.7424 | 0.0209 |
| ***o__Campylobacterales*** | HA | 2.1688 | 0.0274 |
| ***f__Helicobacteraceae*** | HA | 2.1688 | 0.0274 |
| ***c__Epsilonproteobacteria*** | HA | 2.1688 | 0.0274 |
| ***g__Blautia*** | CR | 4.3696 | 0.0016 |
| ***f__Lachnospiraceae*** | CR | 4.1138 | 0.046 |
| ***g___Prevotella_*** | CR | 3.4386 | 0.046 |
| ***o__Erysipelotrichales*** | CR | 2.7925 | 0.0117 |
| ***c__Erysipelotrichi*** | CR | 2.7925 | 0.0117 |
| ***f__Erysipelotrichaceae*** | CR | 2.7925 | 0.0117 |
| ***g__Allobaculum*** | CR | 2.6238 | 0.0117 |
| ***c__4C0d_2*** | CR | 2.0025 | 0.0252 |
| ***c__4C0d_2.o__YS2*** | CR | 2.0025 | 0.0252 |

**Table S5.** Significantly changed pathways after HA. Pathway activity analyses were predicted with both mummichog and GSEA methods using MetaboAnalystR.

| **Pathway Name** | **Total Hits** | **Hits** | **Sig_Hits** | ***P*-value** |
| --- | --- | --- | --- | --- |
| Glycine, serine and threonine metabolism | 32 | 14 | 13 | 2.80E-04 |
| Lysine degradation | 20 | 7 | 7 | 0.0016 |
| Tyrosine metabolism | 42 | 15 | 15 | 0.0086 |
| Aminoacyl-tRNA biosynthesis | 67 | 15 | 12 | 0.0111 |
| Steroid hormone biosynthesis | 70 | 13 | 9 | 0.0151 |
| Steroid hormone biosynthesis | 70 | 15 | 13 | 0.0163 |
| Nicotinate and nicotinamide metabolism | 13 | 2 | 2 | 0.0168 |
| Glycerolipid metabolism | 18 | 4 | 3 | 0.0187 |
| Amino sugar and nucleotide sugar metabolism | 37 | 17 | 5 | 0.0245 |

# Supplementary figures


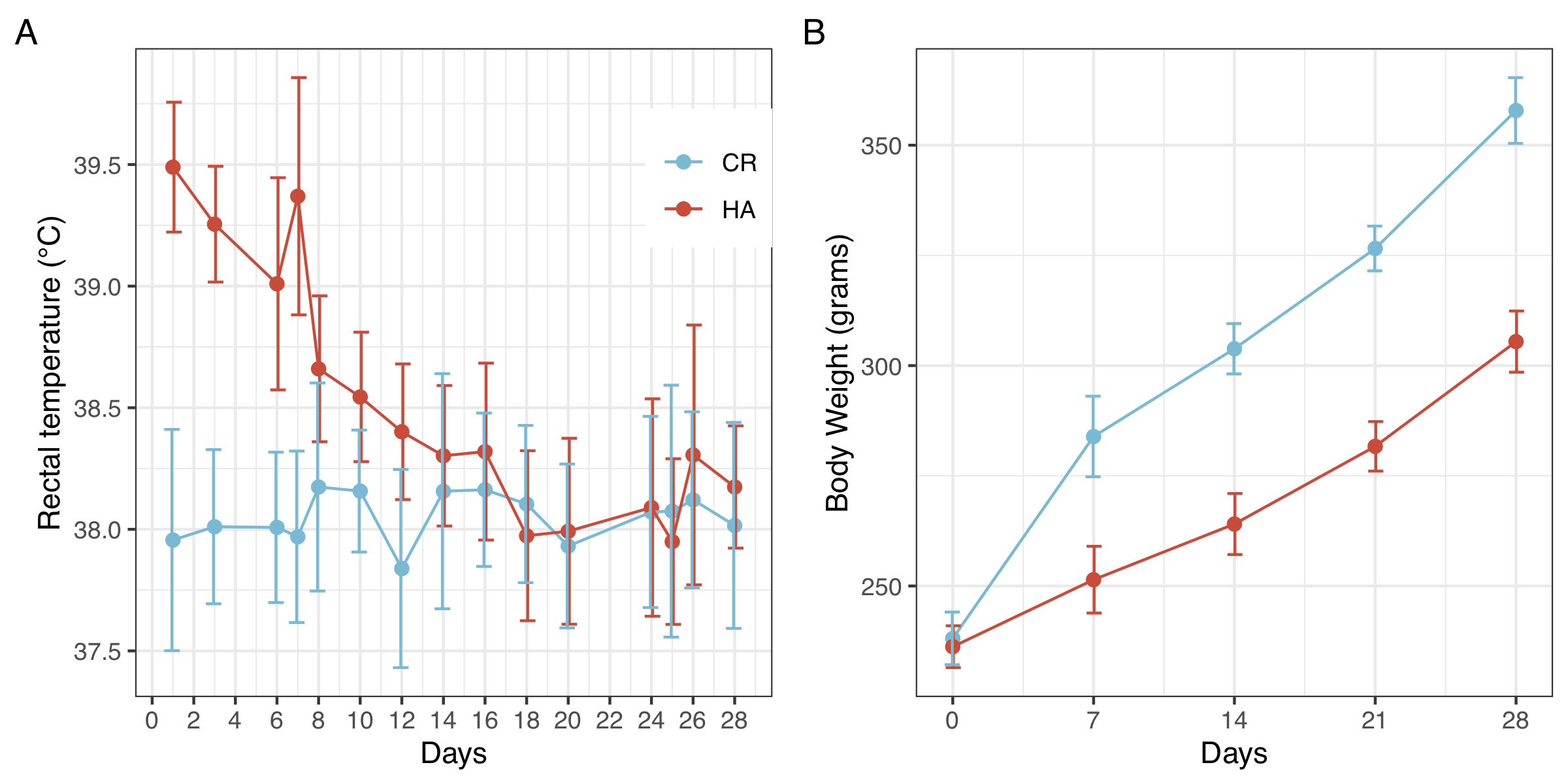


**Figure S1.** Mean rectal temperature **(A)** and body weight **(B)** during 28 days of heat exposure.

**Figure S2.** Quality control of 16S rRNA V3-V4 reads. Number of OTUs **(A)** after quality filtering on day 0, 14, and 28. Wilcoxon test was used to compare CR and HA. **(B)** Rarefaction curves for all samples with the X axis representing the number of sequences and the Y axis representing the number of observed taxa. **(C)** The number of OTUs from the CR group on day 0, 14, and 28. Venn diagram showing the number of OTUs exclusively identified in each group on day 0 **(D)**, 14 **(E)**, and day 28 **(F)**. *P* value: **P* < 0.05; ns, no significance *P* > 0.05.

**Figure S3.** Diversity analysis on day 0. **(A)** Alpha diversity assessed by richness (ACE, Observed) and diversity (Shannon, Simpson). Boxes represent the interquartile ranges, and the inside black plots represent the median and circles are outliers. *P* values are from Wilcoxon rank sum test. Beta diversity assessed by principal coordinate analysis (PCoA) based on the Bray-Curtis **(B)** and Jaccard **(C)** distances. *P* values are from Wilcoxon rank sum test. *P* values: ns, no significance *P* > 0.05.

**Figure S4.** Diversity analysis on day 14. **(A)** Alpha diversity assessed by richness (ACE, Observed) and diversity (Shannon, Simpson). Boxes represent the interquartile ranges, and the inside black plots represent the median and circles are outliers. *P* values are from Wilcoxon rank sum test. Beta diversity assessed by principal coordinate analysis (PCoA) based on the Bray-Curtis **(B)** and Jaccard **(C)** distances. Significant *P*-values of Anosim and multi-response permutation procedure (MRPP) between groups emphasize the differences in microbial community structure. **(D)** Relative abundance of bacterial phyla. *P* values: ng, no significance *P* > 0.05.

**Figure S5.** The degrees of OTUs in the four significant different genera of inferred ecological networks. *P* values are from Wilcoxon rank sum test. *P* value: **P* < 0.05; ns, no significance *P* > 0.05.

**Figure S6.** Targeted metabolomics profiling of celastrol. *P* values are from Wilcoxon rank sum test. *P* value: **P* < 0.05.
